# Densely Carboxylated Graphene for Synthesis of High-Performing NASICON Cathodes for Na-Ion Batteries

**DOI:** 10.1021/acsami.5c21272

**Published:** 2026-01-14

**Authors:** Ievgen Obraztsov, Anita Cymann-Sachajdak, Kamila Bruniecka, Piotr Madajski, Veronika Šedajová, Grzegorz Trykowski, Aristides Bakandritsos, Monika Wilamowska-Zawłocka

**Affiliations:** † Regional Centre of Advanced Technologies and Materials (RCPTM), Czech Advanced Technology and Research Institute (CATRIN), 48207 Palacký University Olomouc, Šlechtitelů 27, Olomouc 77 900, Czech Republic; ‡ Department of Energy Conversion and Storage, Faculty of Chemistry, Gdansk University of Technology, Narutowicza 11/12, 80-233 Gdańsk, Poland; § Hanse-Wissenschaftskolleg, Institute for Advanced Study, Lehmkuhlenbusch 4, 27 753 Delmenhorst, Germany; ∥ Department of Chemistry of Materials, Adsorption and Catalysis, Faculty of Chemistry, Nicolaus Copernicus University in Torun, 7 Gagarina, 87-100 Toruń, Poland; ⊥ Nanotechnology Centre, Centre of Energy and Environmental Technologies, VŠB-Technical University of Ostrava, 17. listopadu 2172/15, 708 00 Ostrava-Poruba, Czech Republic

**Keywords:** sodium, cathode, NASICON, carbon, core−shell, graphene acid, N-doped graphene

## Abstract

Sodium-ion batteries are emerging as a promising alternative to lithium-ion technology due to the abundance and low cost of sodium. Among the cathode candidates, Na_3_V_2_(PO_4_)_3_ (NVP) with a NASICON framework and its analogues offer a high operating voltage and excellent structural stability. However, their practical use is limited by poor electronic conductivity, a low active material fraction, and trade-offs in terms of morphology and tap density. Here, we report a simple synthesis strategy that employs densely carboxylated graphene, graphene acid (GA), as a multifunctional additive. GA acts simultaneously as a chelating agent, pH regulator, and in situ-formed carbon shell prior to calcination. GA allows the efficient reduction of V^5+^ to electrochemically active V^3+^, phase-pure NVP formation, and the growth of a thin, conformal carbon shell strongly anchored to NVP particles. The resulting electrodes contain 85 wt % active material while maintaining outstanding charge-transfer kinetics. The optimized NVP@GA cathode delivers an excellent rate performance up to 15 A g_EM_^–1^ (151 C), retaining 65.4% of the theoretical capacity of NVP, and stable cycling. This approach provides a versatile route for tailoring NASICON cathodes and can be extended to other phosphate-based systems for high-power sodium-ion batteries.

## Graphical abstract

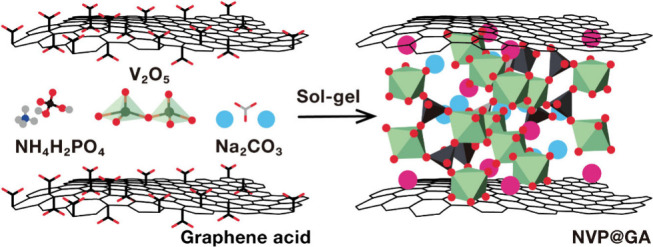

## 1 Introduction

Energy storage is essential for integrating renewable power into the grid and for securing its stability. Lithium-ion batteries (LIBs) dominate the energy storage scene; however, they pose resource supply chain risks.^1,2^ This emphasizes the potential of sodium-ion batteries (SIBs) as a sustainable, affordable, and scalable alternative for large-scale applications where high energy density is less essential.^3,4^ Cathode development remains a key challenge for SIBs; a range of chemistries have been explored, including layered oxides,^5^ Prussian blue analogues,^6^ chalcogens,^7,8^ organic cathodes,^9^ and polyanionic Na superionic conductors (NASICON).^10,11^ Among these, NASICON cathodes offer the best balance of voltage, safety, capacity, and durability.^12^ In particular, Na_3_V_2_(PO_4_)_3_ (NVP) has become a reference NASICON material, providing a flat ∼3.4 V plateau, a specific capacity of 117 mAh g^–1^, and a theoretical energy density of ∼400 Wh kg^–1^,^13^ and is promising as a broad-temperature-range cathode material.^14^ Despite these advantages, the practical implementation of NVP is limited by (i) the intrinsically low electronic conductivity (σ ≈ 10^–6^–10^–8^ S cm^–1^), (ii) the low active material (AM) fraction in electrodes, (iii) morphology constraints that trade particle size against side reactions, and (iv) synthetic challenges of stabilizing V^3+^ and achieving phase purity.^15,16^ Substantial efforts have been undertaken to improve the performance. Several strategies have been proposed to address these issues. Morphology engineering, such as nanoflakes, hollow microspheres, or hierarchical architectures, shortens Na^+^ diffusion paths and buffers structural stress.^17^ From the perspective of the AM, the focus of advancing NASICON cathodes is reducing or replacing vanadium with benign, earth-abundant ions while maintaining structural integrity and favorable Na^+^ transport. For validation of performance, low-vanadium polyanion cathodes require testing under practical conditions because excessive vanadium substitution disrupts the ion-conducting structure.^18^ Vanadium in NVP can be substituted either by a single transition-metal (TM) ion^19,20^ or by a multicomponent “cocktail” of different TM ions, incorporated into the NASICON framework forming high-entropy materials.^21,22^ A comparable enhancement can be obtained by partially substituting the phosphate anion within the NASICON framework^23^ or by combining cationic and anionic substitutions to amplify their synergistic effects.^24,25^

A second major approach involves carbon coating to improve the intrinsically low electronic conductivity of NASICON materials, with strategies spanning from thin amorphous shells to engineered graphene and heteroatom-doped carbon frameworks that markedly enhance charge transport.^13,26,27^ A critical factor in all approaches is the choice of chelating/carbonaceous precursors to control the reduction state of vanadium, carbon shell formation, and ultimately the conductivity and electrochemical kinetics of the final material.^28^ Integrating a small fraction of multiwalled carbon nanotubes (MWCNTs) into NVP during solid-state synthesis markedly enhanced Na-ion diffusion, reduced charge-transfer resistance, and yielded a highly stable MWCNT@NVP cathode.^29^ The addition of reductive carbon during the synthesis of NASICON material refined the particle morphology, stabilized the structure, and improved the capacity and long-term cycling performance.^30^ A uniformly distributed N-doped carbon network further dramatically enhanced the electronic conductivity and the Na^+^ diffusion of NVP over regular carbon, which was reflected in a much better rate performance and ultralong cycling life.^31^ Carbon nanotube-decorated NVP microspheres obtained by spray-drying and carbothermal reduction processes exhibited much less polarization and lower charge-transfer resistance and ohmic resistance than NVP without CNTs. This resulted in much higher capacity values at high current rates.^32^ Although carbon coating is essential, excessive or poorly distributed carbon layers can block Na^+^ transport and dilute AM. Therefore, most reported electrodes contain 60–70% NASICON active phase and up to 30% carbon, far below the ≥95% active content typical in industrial cathodes (for LIBs).^33,34^ This gap underlines the need for scalable synthetic strategies that produce thin, uniform, and conductive shells while maximizing the AM fraction. Therefore, precise control of the carbon shell thickness and its distribution is crucial.^26^

Graphene acid (GA), a densely carboxylated graphene derivative, offers unique advantages.^35^ Its high density of carboxyl groups makes it an efficient chelator for multivalent cations,^36^ while radical sites on its sp^2^ backbone provide additional coordination sites.^36,37^ In this work, we demonstrate GA as a multifunctional additive for NVP synthesis, simultaneously serving as a chelating and reducing agent and as an in situ-formed, highly conducting carbon shell prior to calcination. This strategy enables the full reduction of V^5+^ to V^3+^, the formation of phase-pure NVP, and the growth of a conformal turbostratic carbon shell strongly bonded to NVP particles. The resulting NVP@GA cathode exhibited superior rate performance compared to the sample synthesized with ascorbic acid, despite having a similar carbon content, and maintained excellent electrochemical performance even at high electrode loadings of 85 wt % active material. Furthermore, it outperformed the reference material prepared with high-density N-doped graphene, with the performance advantage of NVP@GA becoming increasingly pronounced at higher current rates. This study explores the multifunctional role of functionalized carbons in tailoring NASICON-type cathodes and outlines a broadly applicable approach to the development of high-power sodium-ion batteries.

## 2 Experimental Section

Fluorographite, DMF, 1-methyl-2-pyrrolidinone (NMP), poly­(vinylidene fluoride) (PVDF), ascorbic acid (AA), V_2_O_5_, NH_4_H_2_PO_4_, Na_2_CO_3_, and Whatman glass microfiber filters were purchased from Sigma-Aldrich. Sodium metal (99.9%) was obtained from Alfa Aesar. The electrolyte was 1 M NaPF_6_ in ethylene carbonate:diethyl carbonate (EC:DEC, 3:7) with 5 vol % fluoroethylene carbonate (FEC) electrolyte, supplied by E-Lyte Innovations GmbH. Conductive carbon (Ketjenblack EC-600JD) was obtained from AkzoNobel Functional Chemicals BV. All chemicals were used without further purification.

### 2.1 Synthesis of Graphene Derivatives

Graphene acid (GA)^37,38^ and N-doped graphene (NG)^39^ were prepared following reported procedures. Briefly, fluorinated graphite was ultrasonically dispersed in DMF under an inert atmosphere for 4 h. For GA, sodium cyanide was added to the dispersion, which was refluxed at 130 °C for 24 h to yield cyanographene. The product was washed, purified by dialysis, and hydrolyzed with nitric acid to obtain an aqueous GA dispersion. Repeated washing and dispersion cycles were used to ensure the purity. For NG, NaN_3_ was added to the fluorographene dispersion, and the reaction was carried out under otherwise identical conditions for 72 h.

### 2.2 Synthesis of NVP@AA, NVP@NG, and NVP@GA Nanocomposites

Three NVP nanocomposites were prepared, NVP@AA, NVP@NG, and NVP@GA, following a reported sol–gel procedure.^40^ Briefly, 4 mmol of V_2_O_5_, 12 mmol of NH_4_H_2_PO_4_, and 6 mmol of Na_2_CO_3_ were dissolved in 70 mL of distilled water under magnetic stirring at room temperature. Subsequently, 6 mmol of AA and 6 mL of polyethylene glycol 400 (PEG-400) were introduced to obtain a green suspension, which was stirred for 30 min and then transferred into a 100 mL Teflon-lined autoclave. The sealed autoclave was maintained at 180 °C for 40 h and cooled naturally to room temperature. The resulting brown product was homogenized by high-power ultrasonication (two times for 5 min). The obtained brown sol was dried at 120 °C overnight in ambient atmosphere, ground thoroughly, and preheated at 350 °C for 4 h under argon. The preheated sample was reground to a fine powder and finally calcined at 750 °C for 6 h under flowing Ar. For NVP@GA, AA was replaced with 210 mg of GA in aqueous dispersion. For NVP@NG, the same mass of NG was used in place of GA with the addition of 2 mmol of AA to adjust the pH and enable the synthesis.

### 2.3 Material Characterization

The morphology of the samples was examined using scanning electron microscopy (SEM, Hitachi SU6600) and HR-TEM (Tecnai F20 X-Twin). Elemental analysis (CHN) was performed using a Vario Macro elemental analyzer. Additional elemental composition was assessed using SEM with energy-dispersive X-ray spectroscopy (EDX, EVO 15 with a SmartEDX spectrometer). Structural features were characterized by X-ray diffraction (XRD, Philips X’Pert) with Cu Kα radiation and an X’Celerator Scientific detector, and Raman spectra were recorded using a Thermo Scientific DXR3 Raman microscope with a 532 nm laser. A monochromated microfocused low-power Al Kα X-ray source was used for X-ray photoelectron spectroscopy (XPS) using a Thermo Fisher Scientific Nexsa G2 XPS system. The Avantage (Thermo Scientific) software was used to analyze the obtained data using a smart background subtraction and Gaussian–Lorentzian functions for peak fitting.

### 2.4 Electrode Preparation and Cell Assembly

Electrodes were prepared by dispersing the synthesized NVP active material (AM), carbon black (CB), and PVDF binder in NMP at a mass ratio of 85:8:7. The dispersion was mixed in a Thinky mixer (1100 rpm, 30 kPa) for 5 min followed by 5 min of ultrasonication. The slurry was cast onto a 15 μm thick carbon-coated aluminum foil (Cambridge Energy Solutions) with a 100 μm doctor blade gap. Films were dried at 80 °C in an oven for 30 min and overnight in a vacuum oven at 120 °C. Dry films were cut into 15 mm disks, calendered with 5 kN cm^–2^ pressure, weighed, and stored in an Ar-filled glovebox ([O_2_] < 0.2 ppm, [H_2_O] < 0.8 ppm) before use. The electrode material (EM) loading was ∼1.5 mg cm^–2^, and the film thickness was ∼30 μm. The CR2032 and research-grade coin cells (PAT-cell of EL-Cell GmbH) were assembled using sodium metal (15.5 mm diameter), Whatman GF, and 1 M NaPF_6_ EC:DEC (3:7 by mass) with 5 vol % FEC as the counter/reference electrode, separator, and electrolyte, respectively, unless otherwise specified.

### 2.5 Electrochemical Measurements

Cyclic voltammetry (CV) and electrochemical impedance spectroscopy (EIS) measurements were performed using a VSP-3e potentiostat equipped with an EIS module controlled by the manufacturer’s software (BioLogic Science Instruments). Charge–discharge rate and stability tests were performed using a Novonix UHPC system with a dedicated thermostatic chamber (Novonix). All measurements were performed at 25 ± 0.5 °C. The reported capacities and currents are for the electrode mass (EM) unless otherwise specified. EIS measurements were performed in the frequency range of 100 kHz to 0.1 Hz with an amplitude of 10 mV using a three-electrode configuration, and sodium metal was used as the counter and reference electrodes in a PAT-cell (EL-Cell).

## 3 Results and Discussion

Three NVP-based samples were synthesized to evaluate the multifunctional role of graphene acid (GA) as a chelating and reducing agent and an in situ-formed conductive carbon shell. First, NVP@GA was prepared without additional carbonaceous or acidic additives (Figure 1a, Figure S1). Then, NVP@NG was prepared using N-doped graphene, which lacks carboxyl groups, and ascorbic acid was used for the pH adjustment. Finally, conventional NVP@AA was prepared using a common method in which ascorbic acid served as a chelating and reducing agent and a carbon source. The resulting samples exhibited distinct carbon contents of 12.7% (NVP@GA), 6.7% (NVP@NG), and 10.3% (NVP@AA) (Table S1). XRD analysis was performed to verify the structures of the materials. The recorded diffractograms (Figure 1c, Figure S2) showed the pure rhombohedral NASICON-type Na_3_V_2_(PO_4_)_3_ phase (space group *R*3̅*c*) for NVP@GA and NVP@AA, and a main phase of Na_3_V_2_(PO_4_)_3_ with a trace amount of the NaVP_2_O_7_ phase (space group *I*2/*a*) for NVP@NG. The NaVP_2_O_7_ phase has recently been reported as a sodium-ion cathode.^41,42^ However, its presence at trace levels in our material does not confer any electrochemical advantage relative to the dominant Na_3_V_2_(PO_4_)_3_ phase. Sharp diffraction peaks indicate high crystallinity after pyrolysis at 750 °C. Rietveld refinement of NVP@GA gave lattice parameters of *a* ≈ 8.729 Å and *c* ≈ 21.807 Å corresponding to the NASICON NVP structure.^43^ The lattice parameters for NVP@NG and NVP@AA were slightly higher than those for NVP@GA, with the highest values obtained for NVP@AA (Table S2). The crystal structure of NVP@GA obtained from Rietveld refinement was visualized using VESTA (Figure 1b).^44^ Crystallite sizes, estimated by the Scherrer equation,^45^ were 26 nm for NVP@NG, 32 nm for NVP@GA, and 36 nm for NVP@AA, respectively, which may reflect differences in chelating by NG + AA, GA, and AA.

**Figure 1:**
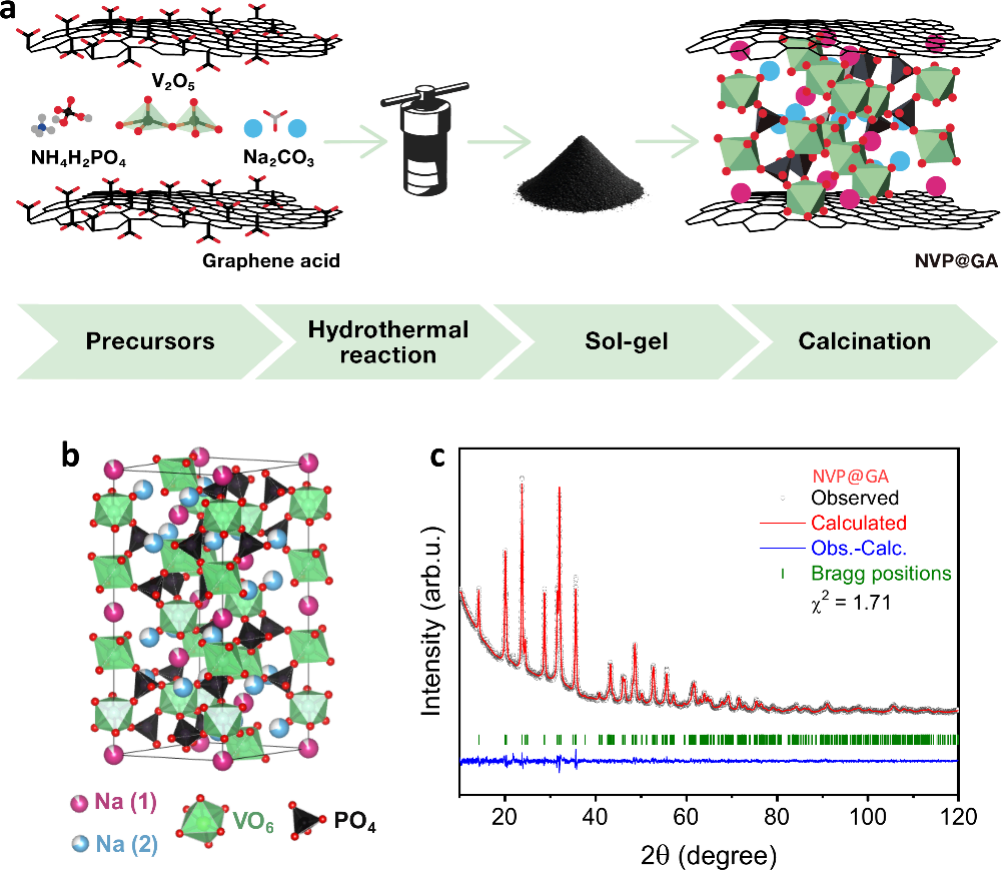
(a) Schematic illustration of the synthesis route, (b) crystal structure of NASICON Na_3_V_2_(PO_4_)_3_, and (c) XRD diffractogram for NVP@GA.

SEM micrographs were collected to examine the morphology of the samples. All materials consisted of NVP nanoclusters encapsulated within carbon (Figure 2a–c). High-resolution transmission electron microscopy (HR-TEM) revealed that NVP@GA had a compact, uniform, ∼10 nm carbon shell of turbostratic carbon that tightly surrounded the NVP crystals (Figure 2d, Figure S3a). In contrast, NVP@NG, which was prepared using N-doped graphene that lacks carboxyl groups, exhibited a nonuniform and partially graphitized carbon coating (Figure 2b,e, Figure S3b). In NVP@AA, carbon formed a heterogeneous composite of large NVP crystals, extended amorphous carbon domains, and dispersed NVP nanoparticles embedded in the carbon matrix rather than in the core–shell (Figure 2c,f). In contrast, NVP@GA showed a homogeneous distribution of the constituent elements in elemental mapping by SEM with energy-dispersive X-ray spectroscopy (Figure 2g). These comparisons show that combining a preformed, conductive shell of densely carboxylated graphene with structural vacancies creates a superior coating that has a favorable structure for Na-ion diffusion, is more uniform, and is better connected to NVP.

**Figure 2:**
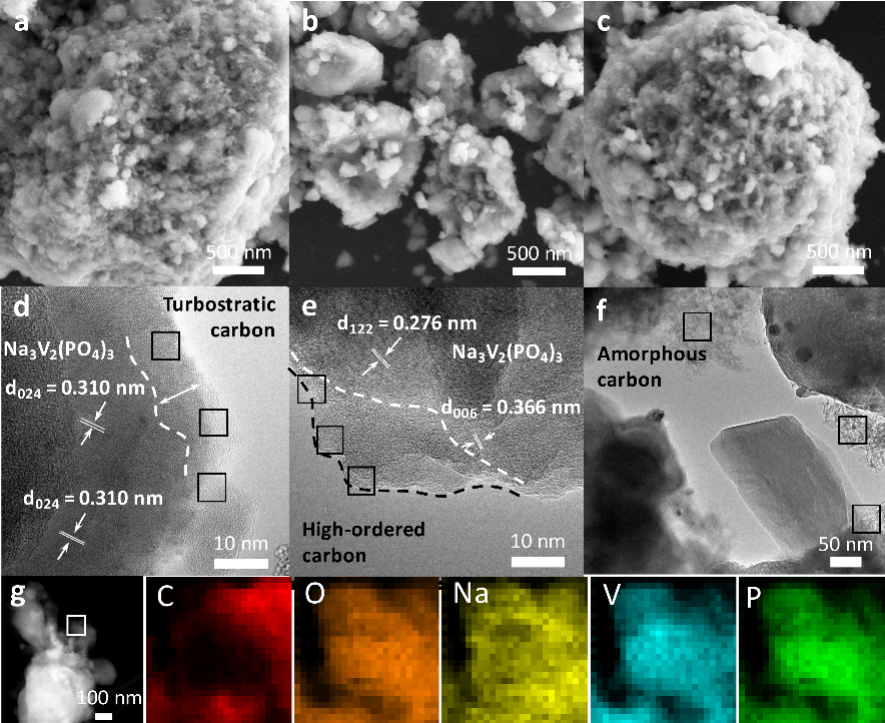
SEM and HR-TEM micrographs of (a, d) NVP@GA, (b, e) NVP@NG (a reference sample without carboxyl groups), and (c, f) NVP@AA (a conventional NVP synthesized using ascorbic acid as the carbon source); (g) elemental mapping of NVP@GA showing the presence and uniform distribution of Na, P, V, O, and C atoms.

X-ray photoelectron spectroscopy (XPS) was used to analyze the surface chemistry of the NVP samples at depths of 2–5 nm.^46^ The survey spectra showed signals corresponding to Na 1s, V 2p, P 2s and P 2p, O 1s, C 1s, and N 1s (Figure S4). These signals are consistent with the composition of the NVP@carbon composites. The high-resolution spectra of each element were deconvoluted to understand the chemical environment of elements (Figure 3, Figure S5). For NVP@GA, the V 2p spectrum exhibited the characteristic V 2p_3/2_ and V 2p_1/2_ doublet of V^3+^ at 516.26 and 523.13 eV, along with V^4+^ contributions at 516.87 and 524.37 eV, respectively (Figure 3a). Quantitative analysis revealed NVP@GA contained 59 at. % V^3+^ and 41 at. % V^4+^. In contrast, NVP@AA showed the opposite ratio, with V^4+^ as the dominant species. NVP@NG contained 33 at. % V^3+^ and 67 at. % V^4+^. These results suggest that the compact carbon shell derived from GA protects the NVP surface from oxidation more effectively. The P 2p spectra showed the typical phosphate doublet with P 2p_3/2_ and P 2p_1/2_ peaks at 132.67 and 133.57 eV, respectively (Figure 3b). For NVP@GA and NVP@AA, these peaks were shifted by ∼0.2 eV toward higher binding energies. Additionally, trace amounts of carbon-bound phosphate were detected in NVP@GA and NVP@AA. Notably, this contribution was substantial in NVP@AA, where a distinct P 2p_3/2_ and P 2p_1/2_ doublet appeared at 134.86 and 135.83 eV, respectively. For NVP@GA, the O 1s spectrum exhibited a peak at 530.45 eV, corresponding to oxygen bonded to V^3+^, and a higher-energy peak at 530.85 eV, associated with O–V^4+^ (Figure 3c). The relative intensities of these peaks matched the V^3+^/V^4+^ ratio determined from the V 2p spectrum. An additional feature at 532.19 eV was assigned to O–C bonds arising from interactions between NVP and the carbon shell. This contribution was most pronounced in NVP@AA, while it was the smallest in NVP@GA. Typically, NVP exposes oxygen-terminated sites from the VO_6_ octahedra or PO_4_ tetrahedra, enabling the carbon shell to anchor through C–O–V and C–O–P linkages formed during the dehydration of carbonaceous precursors and partial reduction of surface oxides during carbonization.^13,17^ By contrast, the relatively weak O–C signal in NVP@GA indicates that its carbon shell is coupled to the NVP surface through a combination of C–O–V and C–O–P linkages, π–π stacking, and van der Waals interactions, rather than solely covalent bonding.^13,47^

**Figure 3:**
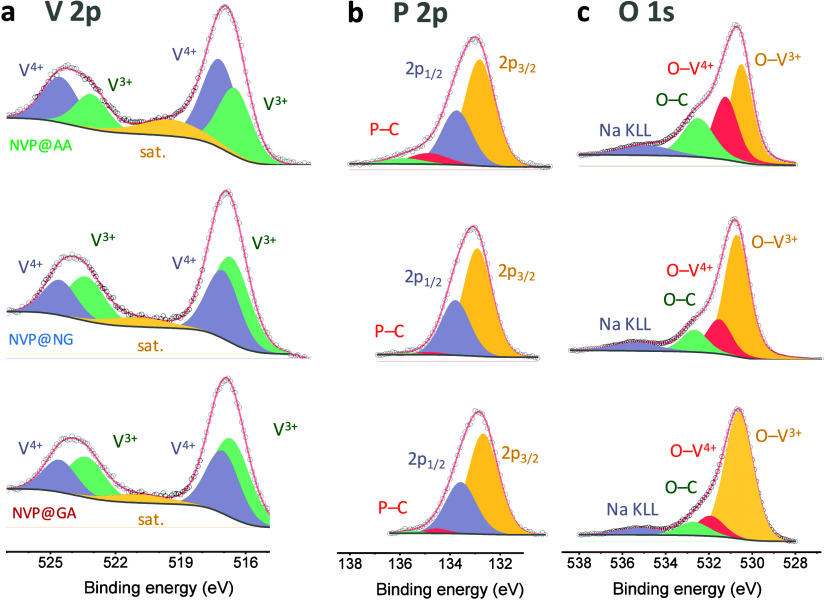
High-resolution XPS spectra and corresponding peak deconvolution of NVP@GA, NVP@NG, and NVP@AA samples for (a) V 2p, (b) P 2p, and (c) O 1s regions.

Raman spectroscopy was conducted to further assess the carbon structure, complementing the XPS and HR-TEM findings (Figure 4). Deconvolution of the D and G bands, following the work of Sadezky et al.,^48^ revealed that the D1/G ratio was nearly identical for NVP@GA and NVP@NG but higher for NVP@AA (Figure 4b, Table S3). The D4/G ratio was comparable across all samples, whereas the D3/G ratio for NVP@AA was approximately twice that of NVP@GA and NVP@NG (Figure 4b), indicating a greater fraction of amorphous carbon. In contrast, NVP@NG exhibited a higher degree of graphitic carbon than NVP@GA, along with a slightly reduced content of amorphous carbon. The average carbon cluster sizes (*L*_a_) were 4.38 nm for NVP@GA, 4.20 nm for NVP@NG, and 3.82 nm for NVP@AA (calculated according to the method of Tuinstra and Koenig,^49^ Note S1). To analyze the carbon shell in more detail, XPS C 1s spectra deconvolution was performed (Figure S5). It revealed that the fraction of highly conductive sp^2^ carbon in the shell was the highest in NVP@GA, followed by NVP@NG and NVP@AA, being 70.8%, 66.7%, and 61.0%, respectively (Figure 4c). The C–O/C–N component remained nearly constant at ∼16 at. % across all samples, while the C=O fraction increased from 5.1 at. % for NVP@GA to 10.8 at. % for NVP@AA. Similarly, the sp^3^ carbon content increased from 3.7 at. % for NVP@GA to 5.9 at. % for NVP@AA. These results collectively indicate that GA-assisted synthesis produces a high-quality carbon shell, characterized by the largest proportion of conductive sp^2^ carbon and the lowest contributions from less conductive carbon species. At the same time, the ascorbic-acid-derived route yields less ordered, partially amorphous carbon coatings.

**Figure 4:**
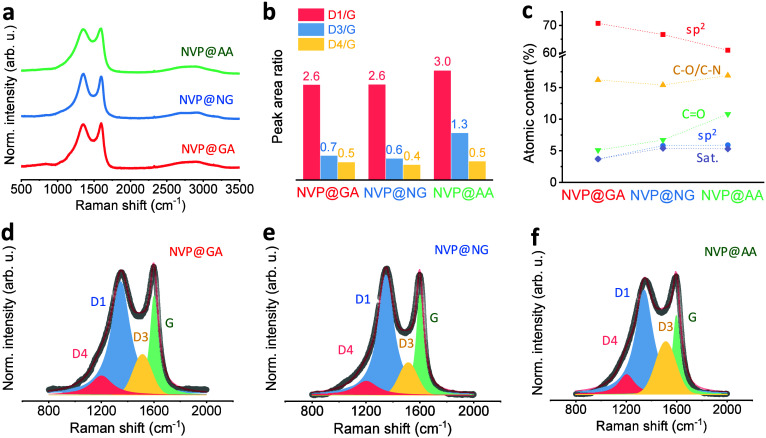
(a) Raman spectra of the NVP samples; (b) peak area ratio of the deconvoluted D and G bands; (c) relative proportions of carbon species derived from XPS C 1s spectra deconvolution; and deconvoluted Raman spectra of the D and G bands for (d) NVP@GA, (e) NVP@NG, and (f) NVP@AA.

A series of electrochemical measurements was carried out to assess the effect of the GA-derived carbon shell on electrochemical performance. Initial cyclic voltammograms (CVs) of the NVP samples showed the characteristic V^4+^/V^3+^ redox couple at ∼3.4 V vs Na/Na^+^ (Figure 5a, Figure S6). NVP@GA and NVP@NG exhibited a single anodic peak at ∼3.46 V, accompanied by split cathodic peaks at ∼3.33 and ∼3.28 V, while NVP@AA displayed an anodic peak at ∼3.50 V and cathodic peaks at ∼3.28 and ∼3.20 V. This splitting of the reduction peak is attributed to rearrangements in the local redox environment during Na^+^ insertion into the Na(1) and Na(2) sites.^50^ The anodic and split cathodic peak separation was ∼110 mV/∼160 mV for both NVP@GA and NVP@NG and increased significantly to ∼220 mV/310 mV for NVP@AA. This indicates substantially higher electronic conductivity in the GA- and NG-derived carbon shells than in the AA-derived coating and typically reported 170–410 mV values.^51–54^ Furthermore, the trend in cathodic peak intensity differed: in NVP@GA and NVP@NG, the higher-potential reduction peak dominated; in NVP@AA, however, the lower-potential peak was more pronounced (Figure 5a, Figure S6). These observations suggest that differences among the samples arise from conductivity and variations in the structural characteristics of the carbon shell and its interaction with NVP. Subsequently, the rate capability was evaluated in half-cells vs Na within the 0.05–15 A g_EM_^–1^ current density range, corresponding to approximately 0.5 C–151 C at the AM level (Figure 5b–d). The NVP@GA cathode delivered 90 mAh g_EM_^–1^ at 0.05 A g_EM_^–1^ (0.5 C), 80 mAh g_EM_^–1^ at 1 A g_EM_^–1^ (10 C), and ∼60 mAh g_EM_^–1^ at 15 A g_EM_^–1^ (151 C). In contrast, NVP@AA exhibited only ∼60 mAh g_EM_^–1^ at 0.05 A g_EM_^–1^ and rapidly faded to 35 mAh g_EM_^–1^ at 1 A g_EM_^–1^. Notably, the capacities of both electrodes fully recovered when the current density was lowered, confirming excellent reversibility and structural stability. Although theoretical capacities of NVP are frequently reported, direct comparison across studies is challenging because the actual NVP content in composite electrodes varies widely (20–70%).^26^ In this work, the actual inorganic AM content in the EM was 73% based on the selected EM composition and NVP@GA CHN analysis (Table S1). The corresponding theoretical capacity of the EM was 85.4 mAh g_EM_^–1^, assuming a 117 mAh g^–1^ theoretical capacity of NVP. The measured capacities of NVP@GA were 90–83.2 mAh g_EM_^–1^ in the 0.05–1 A g_EM_^–1^ (0.5–10 C) range, slightly exceeding or matching the maximum theoretical value. Even at 15 A g_EM_^–1^ (151 C), NVP@GA retained 65.4% of the theoretical capacity of the inorganic part (NVP without carbon shell), highlighting its exceptional rate performance. The initial Coulombic efficiency (CE) exceeded 100%, consistent with partially oxidized vanadium at the surface (Figure 3a). The CEs were 101.7%, 101.2%, and 104.3% for NVP@GA, NVP@NG, and NVP@AA, respectively, which are consistent with the V^3+^/V^4+^ ratios determined by XPS. The second-cycle CEs were 98.5% for NVP@GA, 96.2% for NVP@NG, and 96.7% for NVP@AA. The CE increased with cycling and reached 99.8% for NVP@GA by the 15th cycle.

**Figure 5:**
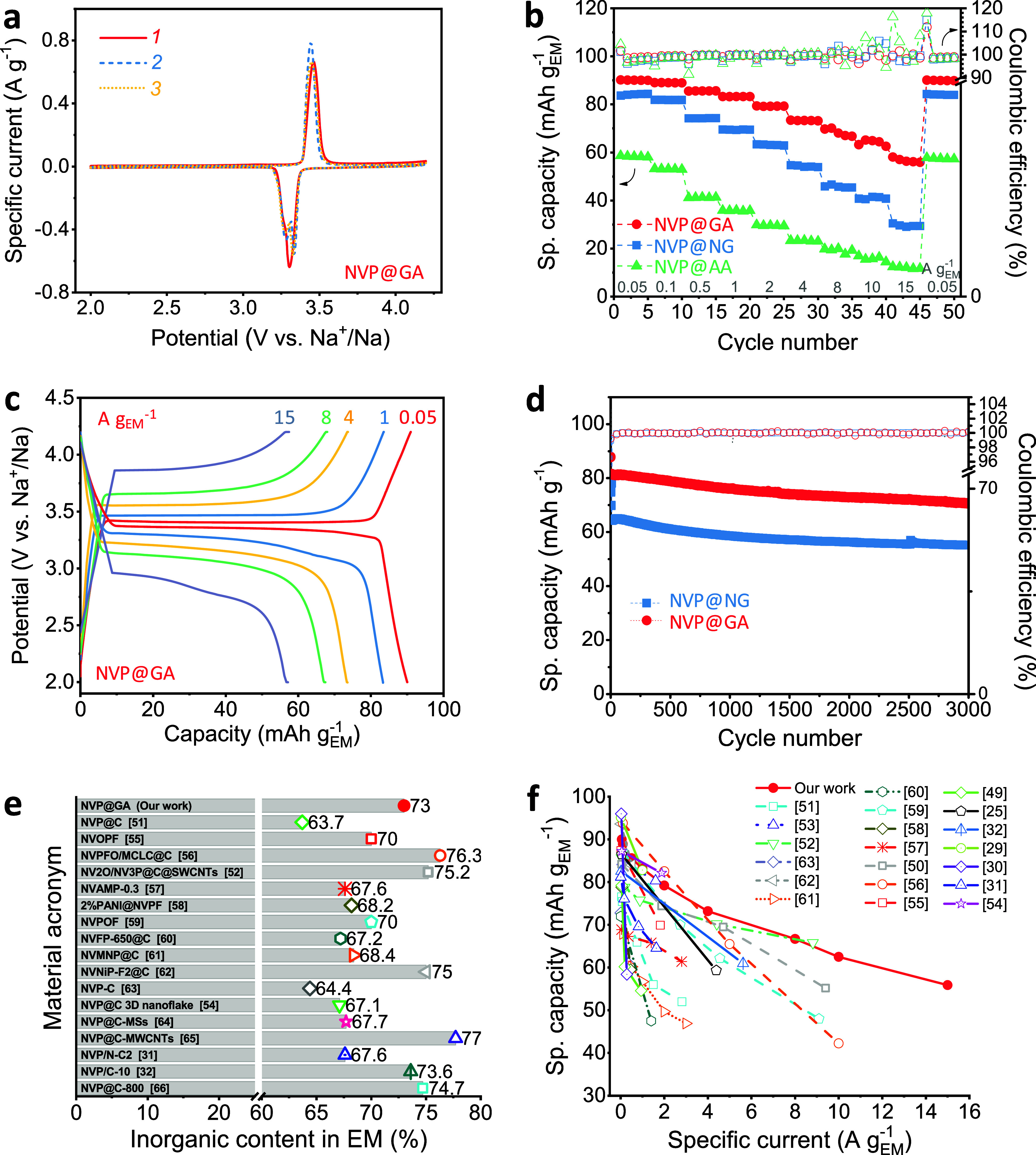
(a) Initial cyclic voltammograms of NVP@GA; (b) rate performance of the NVP cathodes at various specific currents; (c) charge–discharge profiles from the final cycle at each current rate; (d) cycling stability of NVP@GA and NVP@NG cathodes at 1.17 A g_EM_^–1^; (e) comparison of inorganic AM content in EM; and (f) comparison of specific EM capacities with literature-reported data normalized to mass of EM. The references for materials in panels (e) and (f) are NVP@GA (our work), NVP@C,^51^ NVOPF,^55^ NVPFO/MCLC@C,^56^ NV2O/NV3P@C@SWCNT,^52^ NVAMP-0.3,^57^ 2%PANI@NVPF,^58^ NVPOF,^59^ NVFP-650@C,^60^ NVMNP@C,^61^ NVNiP-F2@C,^62^ NVP-C,^63^ NVP@C 3D nanoflake,^54^ NVP@C-MSs,^64^ NVP@C-MWCNTs,^65^ NVP/N-C2,^31^ NVP/C-10,^32^ and NVP@C-800.^66^

To assess the conductivity of the NVP materials, we analyzed the charge–discharge voltage hysteresis. It was 49, 58, and 135 mV at 50% state of charge during cycling at 0.05 A g_EM_^–1^ (Figure 5c, Figure S7), for NVP@GA, NVP@NG, and NVP@AA, respectively. The lower overpotential for NVP@GA, with all other parameters held constant, indicates the higher electronic conductivity of this material. Furthermore, the materials exhibited outstanding cycling stability, with NVP@GA and NVP@NG retaining 87% and 85.6% of initial capacity, respectively, after 3000 cycles at 1.17 A g_EM_^–1^ (∼12 C) (Figure 5d).

Most reported NASICON-based cathodes employ a carbon shell around the AM, and the EM has an inorganic fraction of 60–70% (Figure 5e, Table S4). The remaining is typically carbon or heteroatom additives used to enhance electronic conductivity, and an additional 10–20% carbon black or CNTs is often required because the carbon coating alone is insufficient for optimal cathode performance. In contrast, the NVP@GA composite achieves an inorganic content of 73%, which is among the highest reported values, while requiring only 8% CB in the EM. The GA-derived conformal carbon shell ensures outstanding intrinsic conductivity, allowing the NVP@GA cathode to outperform, at the EM level, not only its direct analogue but also a wide range of NASICON and non-NASICON vanadium-based cathodes (Figure 5f, Table S4).

Kinetic analysis was carried out to clarify the electrochemical mechanism of NVP@GA and NVP@NG. First, multirate CVs were recorded in the 0.2–4.0 mV s^–1^ range (Figure 6a, Figure S8). The *b*-value calculated from the log–log plot of current vs potential sweep rate was 0.57 and 0.60 for NVP@GA and NVP@NG, respectively (Figure 6b), indicating a dominant diffusion-controlled process typical of battery-type behavior (Note S2).

**Figure 6:**
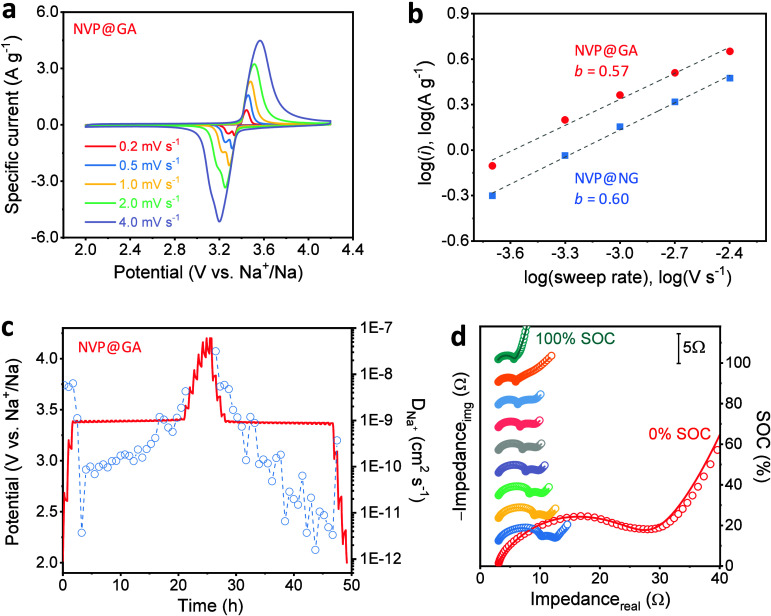
(a) Cyclic voltammetry curves of the NVP@GA cathode at different scan rates; (b) *b*-value analysis of the oxidation peaks in the scan rate range of 0.2–4.0 mV s^–1^; (c) galvanostatic intermittent titration technique (GITT) charge–discharge profile of NVP@GA and the corresponding Na^+^ diffusion coefficients at different states of charge; and (d) Nyquist plots obtained from EIS measurements, recorded galvanostatically for the NVP@GA cathode in a three-electrode cell at selected states of charge.

To evaluate the Na^+^ transport kinetics, GITT measurements were conducted (Figure 6c, Note S3). Representative profiles of pulse and relaxation at open-circuit potential, with marked parameters used for calculating the diffusion coefficient, are shown in Figure S9a (for charge) and Figure S9b (for discharge). The calculated Na^+^ diffusion coefficients (*D*_Na^+^_) fell within the range of ∼2.7 × 10^–12^ to 3 × 10^–9^ cm^2^ s^–1^, consistent with previously reported values for NVP.^64,67^ These results show that the GA shell does not hinder Na^+^ diffusion within the electrode, indicating that the carbon framework enhances the electronic conductivity without impeding ionic transport. To further evaluate charge-transfer behavior, EIS measurements were conducted at different states of charge (Figure 6d, Figure S10a). The Nyquist plots were fitted using two modified Randles circuits (Figure S10b,c). Two distinct semicircles were observed at intermediate states of charge (SOCs). The first resistance element (*R*_1_) varied from 1.6 to 13.5 Ω cm^–2^, and the second element (*R*_2_) ranged from 0.7 to 1.0 Ω cm^–2^, increasing at higher SOCs. Importantly, the second semicircle and *R*_2_ disappeared at 0% and 100% SOC (Table S5). At 0% SOC, vanadium exists entirely in the V^3+^ state, while at 100% SOC, it is fully oxidized to V^4+^. In both cases, no further redox activity is possible, and therefore, no interfacial electron-transfer process takes place. As a result, the charge-transfer resistance vanishes, and the second semicircle disappears from the Nyquist plot. This behavior confirms that the *R*_2_ element of the equivalent circuit is directly associated with the charge-transfer resistance of the NVP@GA cathode. By comparison, reported literature values lay in the 3–150 Ω cm^–2^ range.^68–70^ The exceptionally low charge-transfer resistance values highlight the outstanding electronic conductivity of the NVP@GA composite. At low frequencies, the Warburg region exhibited a slope of ∼45° at low SOCs, which is characteristic of semi-infinite diffusion, while at 100% SOC (fully charged state), there was a finite-length Warburg or capacitive-type behavior with a ∼90° slope. This indicates complete charging of NVP@GA—desodiation of two Na^+^ sites in the NASICON framework—and that no further Na^+^ ions are available for extraction. The *R*_CT1_ shows two distinct regions at different SOCs. At the beginning, *R*_CT1_ decreases sharply from 13.5 to 4.6 Ω cm^–2^ between 0% and 11% SOC. The initial sharp change is accompanied by an equally sharp decrease in *D*_Na^+^_ determined using GITT (Figure S11); the exchange current approximately doubled (Figure S11), and the minimum of *D*_Na^+^_ determined based on the Warburg coefficient connected with the second semicircle of EIS (Figure 11d). These effects are associated with the activation energy of the Na-ion diffusion process. Then, *R*_CT1_ linearly decreases to 1.6 Ω cm^–2^ between 11% and 100% SOC (Figure S11). This improvement of interfacial kinetics is accompanied by the growth of *D*_Na^+^_ determined using GITT (Figure S11) and the exchange current. The *R*_CT1_ may reflect a mixed effects phase transition from rhombohedral Na_3_V_2_(PO_4_)_3_ to triclinic Na_2_V_2_(PO_4_)_3_ as reported by Park et al.^71^ Progressive improvement of Na-ion transport and interfacial ordering is observed, evidenced by rising diffusion coefficients, exchange current, *Q*_1_, and a mid-SOC maximum in *n*_1_, leading to steadily enhanced charge-transfer kinetics and a continuous decline in *R*_CT1_ up to full vanadium oxidation.

## 4 Conclusions

In this study, we developed a simple yet highly effective strategy to overcome the long-standing limitations of NASICON-type cathodes. We achieved this using GA as a multifunctional additive. GA served multiple roles, acting as a chelating agent and an in situ-formed carbon shell prior to calcination. The densely carboxylated, sp^2^-rich framework of GA enabled the efficient reduction of vanadium and the formation of a conformal, highly conductive carbon shell that was tightly anchored to NVP particles. This architecture significantly enhances electronic conductivity while maintaining a high electrode density. Consequently, the GA shell allowed NVP to exhibit its theoretical capacity in the 0.05–1 A g_EM_^–1^ range and retain 65.4% of it even at 15 A g_EM_^–1^ (∼151 C). The NVP@GA showed good cycling stability, maintaining 87% of its initial capacity after 3000 cycles at 1.17 A g_EM_^–1^. Compared to other reported materials, GA conferred superior electronic conductivity, reducing the charge-transfer resistance to 0.7–1.0 Ω cm^–2^ without hindering Na^+^ diffusion. Furthermore, NVP@GA exhibited an optimized crystallite size and the highest Na occupancy, which likely contributes to its superior performance. In contrast, NVP@NG (the reference sample synthesized with highly conductive N-doped graphene without carboxyl groups) showed considerably lower performance, underscoring the critical role of carboxyl functionality in synthesis and performance optimization. Overall, this work highlights the multifunctional role of GA in simultaneously tuning structure, conductivity, and redox chemistry. Beyond NVP, this strategy provides a broadly applicable route for designing next-generation NASICON-type cathodes, where conformal carbon shells are essential for achieving high conductivity, robust cycling stability, and scalable electrode architectures.

## Supplementary Material



## Data Availability

Data for this article are available on Zenodo and can be found under the same title as in this publication or DOI number (https://zenodo.org/records/17882860).

## Notes

Determination of lateral crystallite size of carbon from Raman spectra; kinetic analysis of cyclic voltammetry data; calculation of Na^+^ diffusion coefficient in GITT experiment; kinetic analysis of charge-transfer processes at the carbon–NVP interface; SEM and TEM micrographs of GA and NG; XRD diffractograms with Rietveld refinement results for reference materials; HR-TEM images with FFT for NVP@GA and NVP@NG; XPS survey spectra of all tested materials; deconvolution of C 1s XPS spectra; initial CVs for NVP@NG and NVP@AA; charge–discharge profiles of NVP@NG and NVP@AA; multirate CVs of NVP@NG; GITT pulses of NVP@GA with indicated parameters for *D*_Na^+^_ calculation; EIS equivalent circuits; interfacial kinetic analysis for NVP@GA; CHN elemental analysis, crystallographic cell parameters; deconvolution results of Raman spectra; performance comparison with literature data; and EIS fitting parameters for NVP@GA at different SOCs (PDF)
