# Supplementary material for: Densely Carboxylated Graphene for Synthesis of High-Performing NASICON Cathodes for Na-Ion Batteries
Source: ACS Appl Mater Interfaces. 2026 Jan 14;18(3):5279–89. doi: 10.1021/acsami.5c21272 (PMC12956273; doi:10.1021/acsami.5c21272)
Supplement: Supplementary file 1 [file am5c21272_si_001.pdf]

Supporting Information

Densely carboxylated graphene for synthesis of high-performing NASICON cathodes for Na-ion batteries

Ievgen Obraztsov,[1*] Anita Cymann-Sachajdak,[2,3] Kamila Bruniecka,[2]
Piotr Madajski,[4] Veronika Šedajová,[1] Grzegorz Trykowski,[4]
Aristides Bakandritsos[1,5] Monika Wilamowska-Zawłocka[2*]

1. Regional Centre of Advanced Technologies and Materials (RCPTM), Czech Advanced Technology and Research Institute (CATRIN), Palacký University Olomouc, Šlechtitelů 27, Olomouc, 77 900, Czech Republic
2. Department of Energy Conversion and Storage, Faculty of Chemistry, Gdansk University of Technology, Narutowicza 11/12, 80-233 Gdańsk, Poland
3. Hanse-Wissenschaftskolleg, Institute for Advanced Study, Lehmkuhlenbusch 4, 27 753 Delmenhorst, Germany
4. Department of Chemistry of Materials, Adsorption and Catalysis**,** Faculty of Chemistry, Nicolaus Copernicus University in Torun, 7 Gagarina, 87-100 Toruń, Poland
5. Nanotechnology Centre, Centre of Energy and Environmental Technologies, VŠB-Technical University of Ostrava, 17. listopadu 2172/15, 708 00 Ostrava-Poruba, Czech Republic

* Corresponding authors' emails:
I.O.: ievgen.obraztsov@upol.cz
M.W.-Z.: monika.wilamowska@pg.edu.pl

Table of SI contents

Note S1. Determination of lateral crystallite size from Raman spectra

Note S2. Kinetic analysis of cyclic voltammetry data

Note S3. Calculation of Na^+ diffusion coefficient in GITT experiment

Note S4. Kinetics of charge transfer over carbon interface

Fig. S1. SEM and TEM micrographs of GA and NG

Fig. S2. XRD diffractograms for reference materials

Fig. S3. HR-TEM images with FFT at the interface

Fig. S4. XPS survey spectra

Fig. S5. Deconvolution of C 1s HR XPS spectra

Fig. S6. Initial CVs for NVP@NG and NVP@AA

Fig. S7. Charge-discharge profiles of NVP@NG and NVP@AA

Fig. S8. Multi-rate CVs of NVP@NG

Fig. S9. GITT pulses of NVP@GA

Fig. S10. EIS equivalent schemes

Fig. S11. Interfacial kinetics analysis for NVP@GA

Table S1. Elemental CHN analysis of the NVP samples

Table S2. Crystallographic cell parameters

Table S3. Deconvolution results of Raman spectra

Table S4. Comparison of NVP cathodes

Table S5. EIS fitting parameters for NVP@GA

Note S1. Determination of lateral crystallite size from Raman spectra

The lateral crystallite size (L_a) of the carbon samples was estimated from Raman spectroscopy based on the Tuinstra and Koenig equation (S1):[1]

$$L_a(nm) = \frac{4.4}{\frac{I_D}{I_G}} \cdot \left(\frac{2.41}{E_L}\right)^4 \quad \text{(S1)}$$

where I(D) and I(G) represent the intensities of the D and G bands, respectively, and E_L is a laser energy, which is equal to 2.3308 eV for 532 nm. Given that the Raman spectra were deconvoluted according to Sadezky et al.,[2] we calculated L_a using the intensity of D1 and G bands.

Note S2. Kinetic analysis of cyclic voltammetry data

To assess the kinetics of NVP cathodes, power-law analysis was employed to elucidate the electrochemical processes during charge and discharge.[3] The b-value, derived from the relationship $i_p = av^b$, was obtained from the slope of the log(i) versus log(v) plot at various potentials. A b-value of 0.5 indicates a diffusion-controlled process, while 1.0 corresponds to a surface-controlled mechanism. Intermediate values observed across most potentials suggest a combination of both.

Note S3. Calculation of Na^+ diffusion coefficient in GITT experiment

The galvanostatic intermittent titration technique (GITT) was carried out in a PAT-Cell (El-Cell) using an NVP@GA cathode and a sodium metal counter electrode. Prior to the measurement, the cathode was conditioned by two charge-discharge cycles at 0.05, 0.1, 0.5, and 1 A g_{EM}^{-1}. The GITT protocol consisted of a sequence of 15 min current pulses at 11.7 mA g_{EM}^{-1}, each followed by a 30-min relaxation at open-circuit potential. The apparent Na^+ diffusion coefficient (D_{Na+}) was determined from the simplified Weppner-Huggins equation, valid for small current perturbations (S2):[4,5]

$$D_{\mathrm{Na+}} = \frac{4}{\pi\tau}\left(\frac{n_{\mathrm{M}}\, V_{\mathrm{M}}}{M_{\mathrm{r}}\, S}\right)^2 \left(\frac{\Delta E_s}{\Delta E_t}\right)^2 \quad \text{(S2)}$$

where τ is the pulse duration (s); n_m and V_m are the molar quantity (mol) and molar volume (cm^3 mol^{-1}) of the active material; M_r is the molar mass (g mol^{-1}); S is the electrode surface area (cm^2); ΔE_t is the potential change during the current pulse (corrected for iR drop); and ΔE_s is the steady-state potential change after relaxation.

Note S4. Kinetic analysis of charge-transfer processes at the carbon-NVP interface

Exchange current density I_0 at SEI/electrolyte interface at different states of charge was calculated based on the following equation (S3):

$$I_0 = \frac{RT}{nFAR_{CT1}} \quad \text{(S3)}$$

where R stands for gas constant (R = 8.314 J mol^{-1} K^{-1}), T – absolute temperature (298.15 K in our case), n – number of transferred electrons, that is one for Na^+, F – Faraday constant (F = 96485 C mol^{-1}), A – geometric surface area of the cathode, R_{CT1} – charge transfer resistance obtained from EIS. High exchange current density represents fast Na^+ exchange through the CEI layer, which is a desirable property, especially at high charge/discharge rates.[6]

Na^+ solid-state diffusion coefficient of Na^+ through SEI (D_{SEI}) at different states of charge was calculated based on the Warburg coefficient ($\sigma = Y_0^{-1}$) (S4):

$$D_{\mathrm{SEI}} = \frac{R^2T^2}{2A^2n^4F^4C^2\sigma^2} \quad \text{(S4)}$$

where C – stands for concentration of Na^+ in the electrolyte (1 mol dm^{-3} in this work).

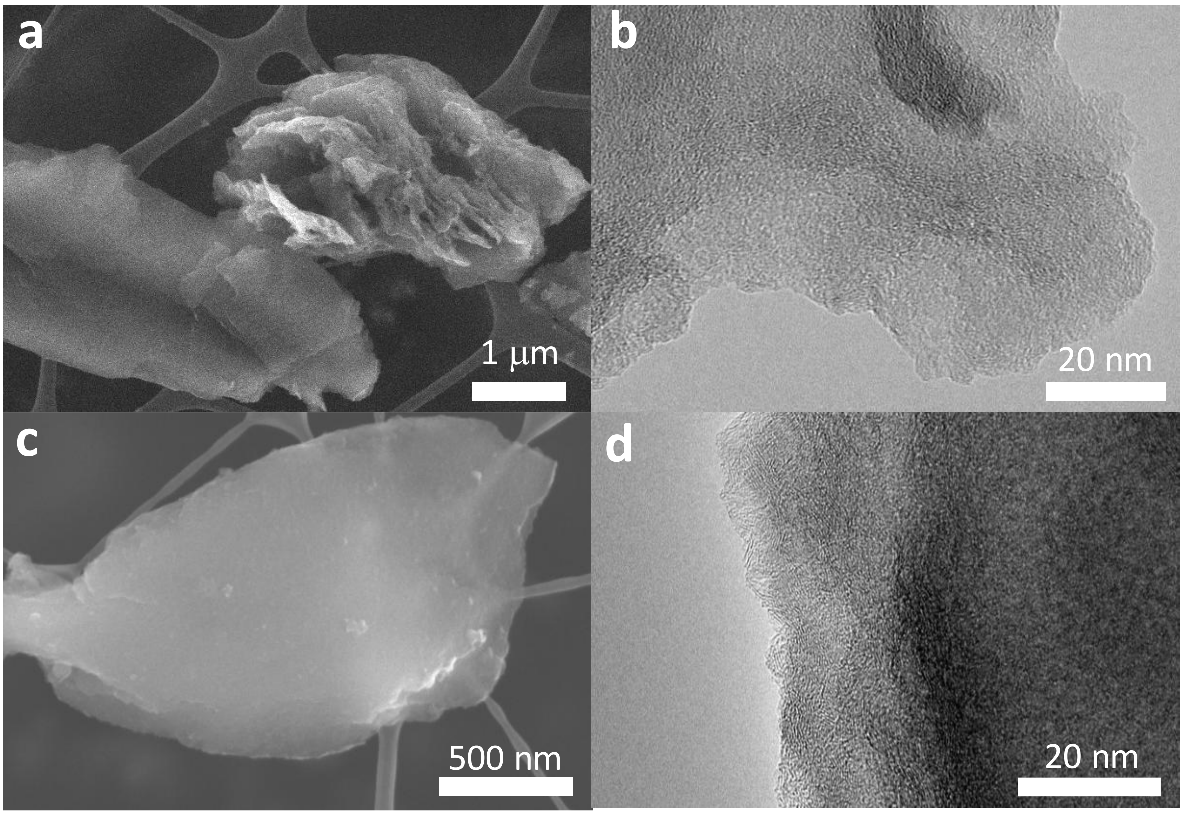

Figure S1. SEM and TEM micrographs of (a,b) GA and (c,d) NG.

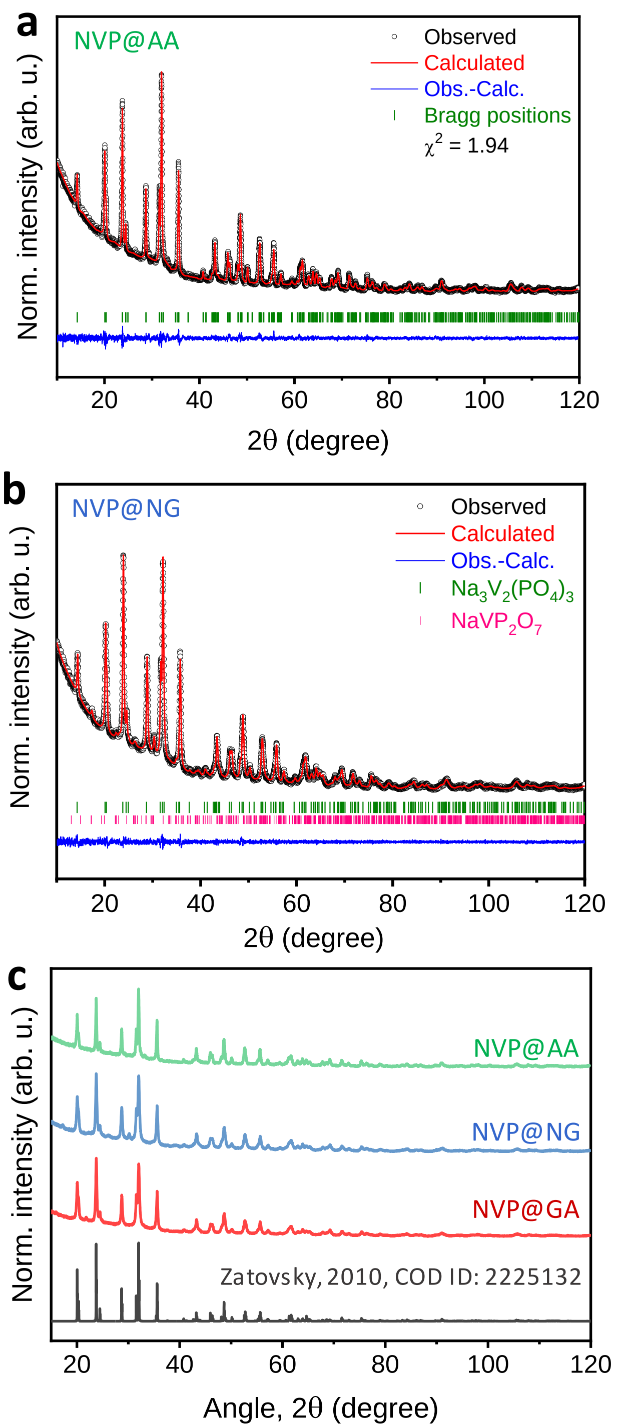

Figure S2. XRD diffractograms for (a) NVP@AA, (b) NVP@NG, and (c) comparison of materials with a reference diffractogram.

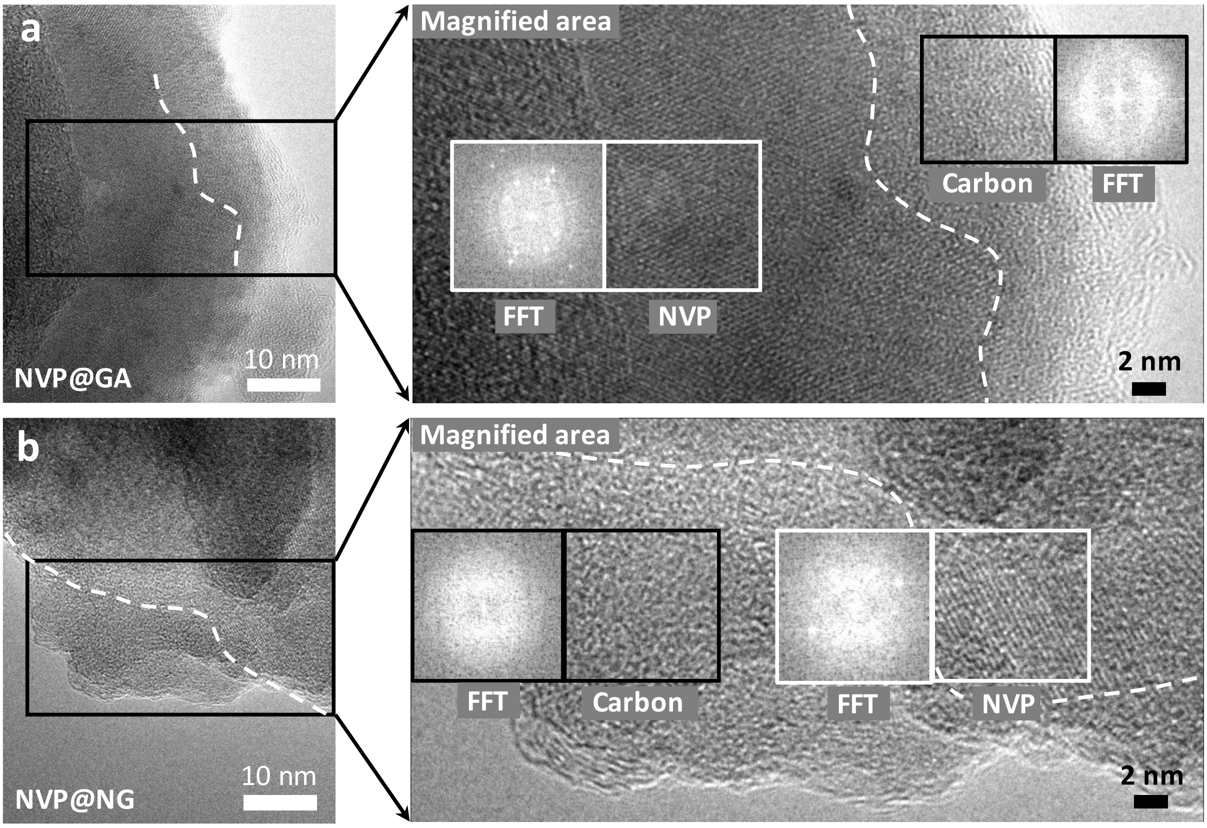

Figure S3. High-resolution TEM images of (a) NVP@GA and (b) NVP@NG with magnified regions at the NVP/carbon interface. Fast Fourier transform (FFT) analyses acquired from the interfacial domains reveal well-defined diffraction spots on the NVP side, indicative of its crystalline order, whereas the carbon regions exhibit no discernible periodicity, consistent with their low-order character.

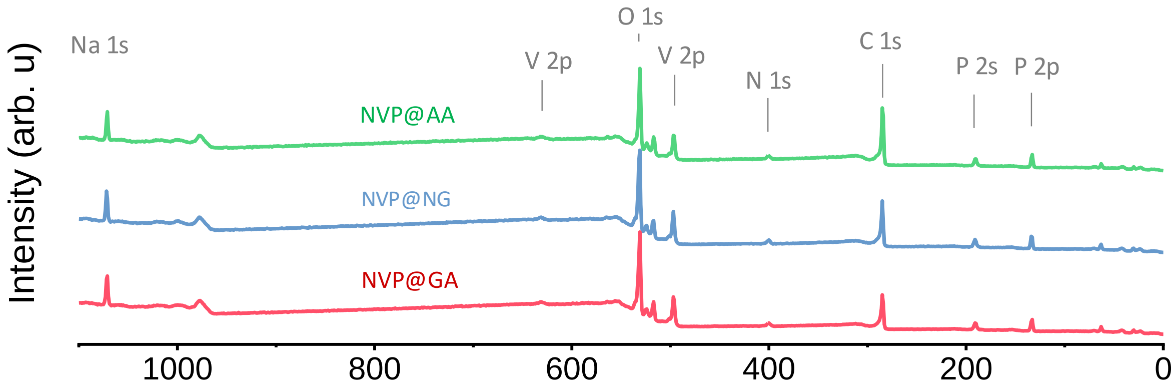

Figure S4. XPS survey spectra for NVP@AA, NVP@NG, and NVP@GA.

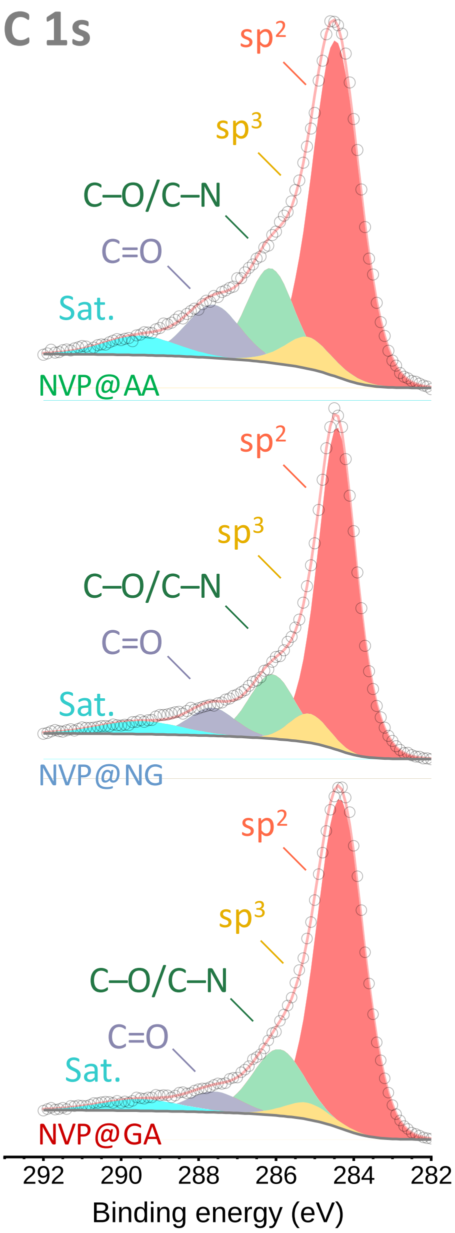

Figure S5. Deconvolution of C 1s high-resolution XPS spectra of NVP samples.

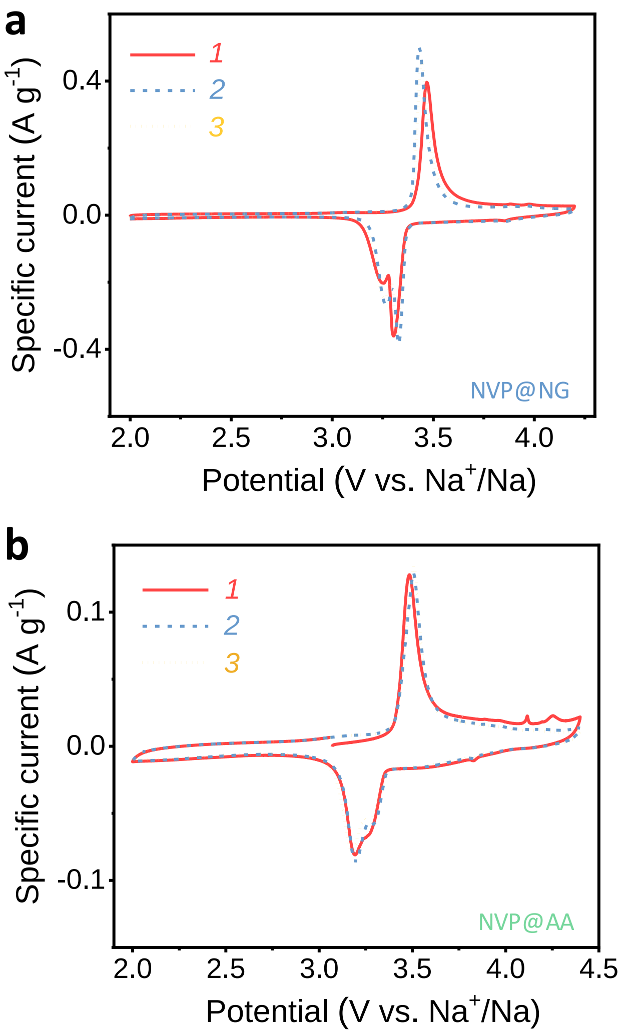

Figure S6. Initial cyclic voltammograms of (a) NVP@NG at 0.2 mV s^{-1} in the 2.0-4.2 V range vs Na/Na^+ and (b) NVP@AA at 0.1 mV s^{-1} in the 2.0-4.4 V range vs Na/Na^+.

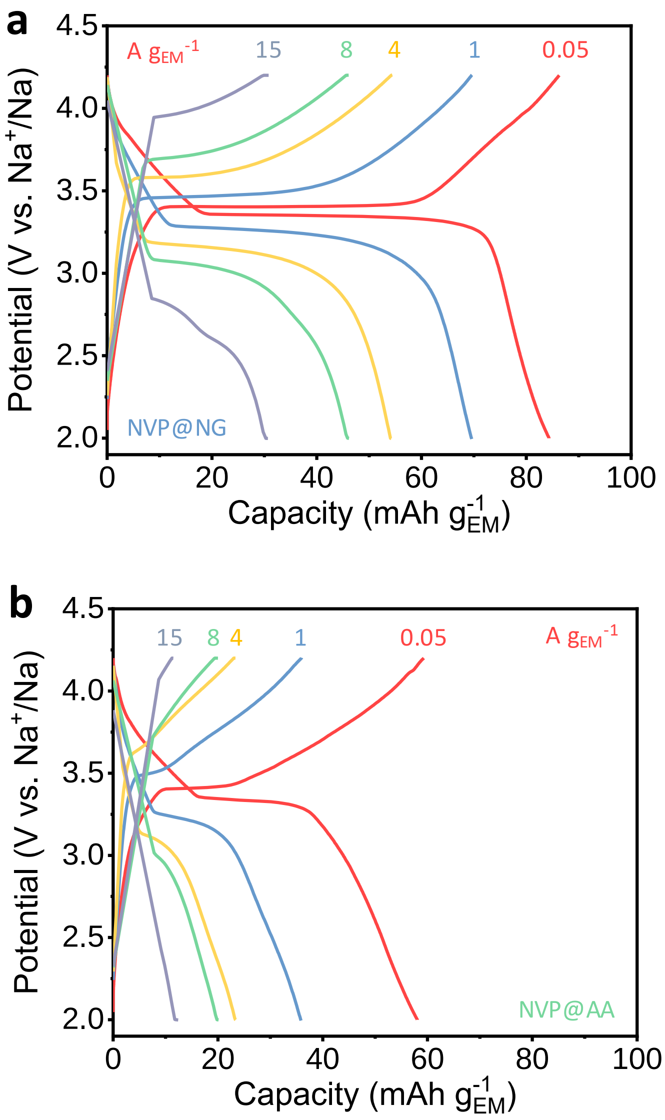

Figure S7. Fifth-cycle galvanostatic charge-discharge profiles of (a) NVP@NG and (b) NVP@AA cathodes, corresponding to the rate performance shown in Fig. 5b.

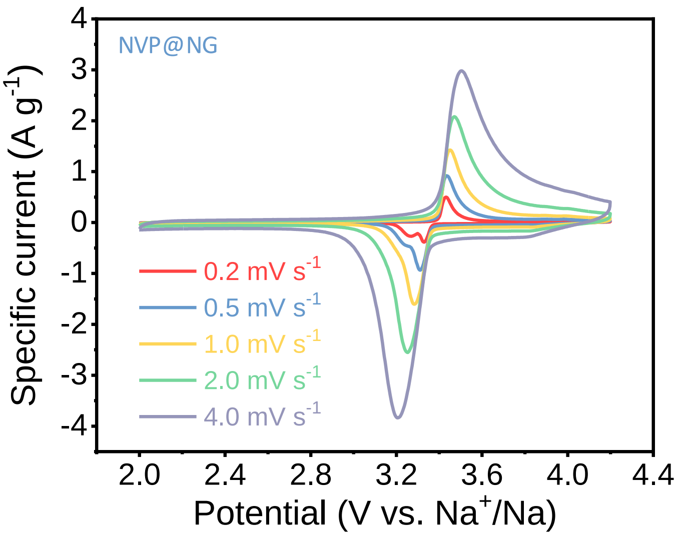

Figure S8. Cyclic voltammetry curves of the NVP@NG cathode at different scan rates.

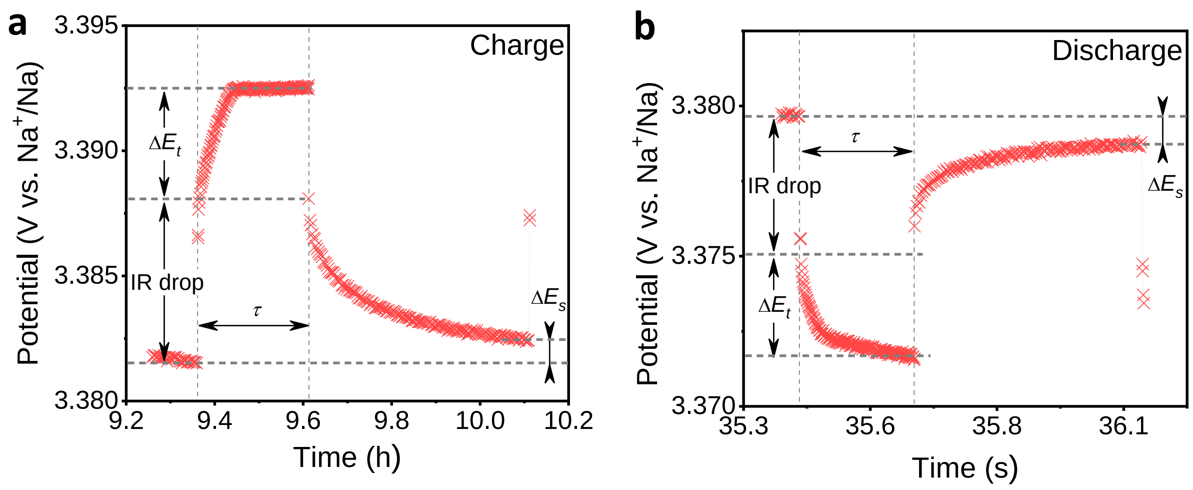

Figure S9. Galvanostatic intermittent titration technique profiles: individual current pulses and relaxation during (a) charge and (b) discharge of NVP@GA.

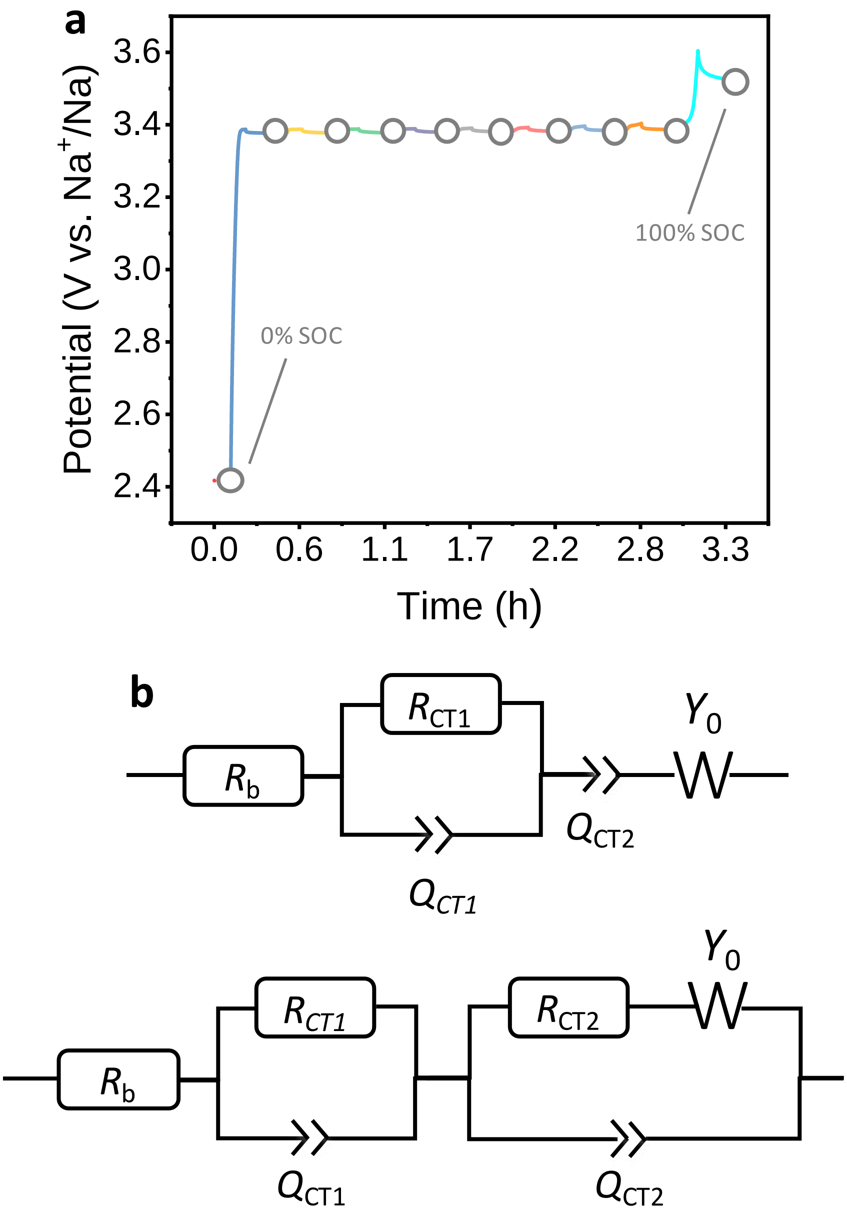

Figure S10. (a) Charge profile of NVP@GA with indicated state of charge where EIS spectra were recorded. Equivalent circuit model used to fit the electrochemical impedance spectra of NVP@GA at the (b) fully sodiated (0% SoC) and desodiated (100% SoC) and (c) at the intermediate states of charge. R_b represents the bulk resistance (electrodes, electrolyte, separator and contacts), the high-frequency semicircle (R_{CT1}, Q_{CT1}) corresponds to the SEI/CEI layer; the mid-frequency arc (R_{CT2}, Q_{CT2}) represents interfacial charge transfer of Na^+ ions; and the low-frequency Warburg element (Y_0) accounts for Na^+ diffusion within the electrode.

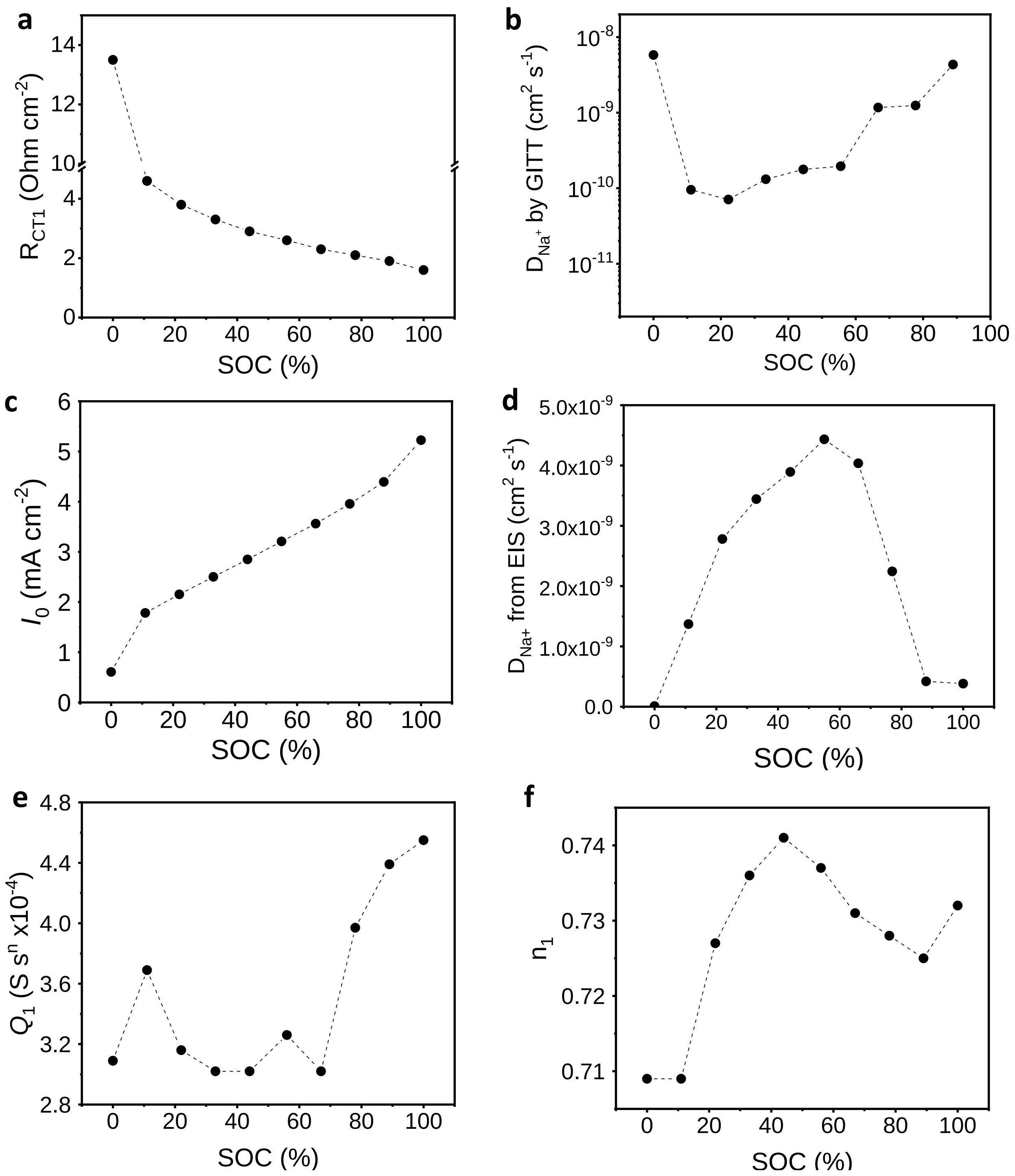

Figure S11. Kinetic analysis of charge transfer across the carbon interface of NVP@GA as a function of state of charge. Shown are: (a) the charge-transfer resistance R_{CT1}, (b) the Na^+ diffusion coefficient obtained from GITT; (c) the exchange current density; and (d) the Na^+ diffusion coefficient extracted from the high-frequency EIS equivalent circuit; (e) the CPE magnitude parameter Q_1; (f) the CPE exponent n_1.

Table S1. Elemental CHN analysis of the NVP samples.

Sample	C	H	N
NVP@GA	12.7	0.6	0.8
NVP@NG*	6.7	0.7	0.5
NVP@AA	10.3	1.0	1.1

* 2 mmol of AA was added to the synthetic mixture of NVP@NG to adjust the pH.

Table S2. Cell parameters for the NVP@GA, NVP@NG, and NVP@AA samples derived from Rietveld refinement.

Sample name	***a*** / Å	***c*** / Å
NVP@GA	8.72929(23)	21.80726(92)
NVP@NG	8.72854(25)	21.81830(99)
NVP@AA	8.73589(14)	21.83198(52)

Table S3. Deconvolution results of Raman spectra for NVP samples presented in Fig 4. I_D/I_G – intensity ratio and A_D/A_G – area ratio of respective bands.

Sample	Peak	Center Max	Area FitTP	Max Height	FWHM	Peak ratio	A_D/A_G	I_D/I_G
**NVP@GA**	D4	1201	10.29	0.154	198.2	D4/G	0.486	0.197
	D1	1347.3	54.54	0.900	175.2	D1/G	2.577	1.148
	D3	1512	14.02	0.324	167.7	D3/G	0.663	0.413
	G	1599.4	21.16	0.784	74.1	–	–	–
**NVP@NG**	D4	1202	9.07	0.107	202.9	D4/G	0.416	0.143
	D1	1348.4	56.36	0.899	143.4	D1/G	2.584	1.197
	D3	1514	12.76	0.246	161.6	D3/G	0.585	0.328
	G	1599.1	21.81	0.751	63.8	–	–	–
**NVP@AA**	D4	1203	8.87	0.161	152.2	D4/G	0.510	0.252
	D1	1342.5	51.53	0.839	169.4	D1/G	2.960	1.318
	D3	1513	22.19	0.426	193.0	D3/G	1.274	0.669
	G	1596.6	17.41	0.637	71.7	–	–	–

Table S4. Comparison of electrochemical properties of NVP cathodes for Na-ion storage. Current and capacity values refer to the mass of electrode material.

%	Active material	Carbon source	EM composition	Inorg. in EM (%)*	Cell	Q_{EM} (mAh g_{EM}^{-1}) @ rate (A g_{EM}^{-1})*	Ref.
1	**NVP@GA 14.1% of CHN by elemental analysis**	**Graphene acid**	**NVP@GA:CB:PVDF 85:8:7**	**73**	**1M $NaPF_6$, EC:DEC (3:7), 5% FEC**	**90.0 @ 0.05 55.9 @ 15**	**This work**
2	NVP@C-800, 6.6% of carbon by TGA	Citric acid	NVP@C800: CB: PVDF 80:10:10	74.7	1M $NaClO_4$ in EC:DEC (1:1), 5% FEC	82.4 @ 0.019 52 @ 2.81	[7]Zhou et al., *ACS AMI* 2021
3	NVP/C-10	Citric acid Carbon nanotubes	NVP/C-10:CB:PVDF 80:10:10	73.6	1 M $NaClO_4$ in EC/DMC (1:1), 5% FEC	82.6 @ 0.0184 60.9 @ 5.62	[8]Chen et al., ACS AMI 2018
4	ME-NASICON	Citric acid Ascorbic acid	ME-NASICON:CB:PVDF 70:20:10	≤70	1 M $NaClO_4$ in PC, 5% FEC	113 @ 0.0084 52.8 @ 1.68	[9]*Zhu* et al., *ACS AMI 2025*
5	KAS_4-NVP	Citric acid	KAS_4-NVP:CB:PVDF 75:20:5	≤75	1 M $NaClO_4$ in EC/DEC (1:1), 5% FEC	86.5 @ 0.044 59.3 @ 4.39	[10]*Chen* et al., *ACS AMI 2025*
6	FNV-2	trisodium citrate dihydrate MWCNTs (0.5wt%)	FNV-2:CB:PVDF 85:5:10	≤85	1 M $NaClO_4$ in EC/DEC (1:1), 5% FEC	95.1 @ 0.01 82.8 @ 1	[8]*Kadam* et al., *ACS AMI 2023*
7	NMTP WC	Citric acid, Acetates, super P, tetraisopropoxide	NMTP WC:CB:PVDF 80:10:10	≤80	1 M $NaPF_6$ in diglyme	96 @ 0.028 58.4 @ 0.28	[11]*Xia* et al., *ACS AMI 2025*
8	NVP/N–C2	Citric acid Glucose, urea	NVP/N–C2:CB:PVDF 70:20:10	67.6	$NaPF_6$ in EC:DMC	81.2 @ 0.0082 77.7 @ 0.082 69.6 @ 0.82 64.54 @ 1.64	[12]*Li* et al., *ACS AMI 2025*
9	NVP@C-MWCNTs, 2.83% of carbon by TGA	Oxalic acid, MWCNTs	NVP@C@MWCNTs: CB: PVDF 80:10:10	77.7	1M $NaPF_6$ in diglyme	84.8 @ 0.08 80.3 @ 1.6	[13] Wang et al., *Chem. Eng. J. 2018*
10	NVP@C-CC, free-standing EM, 8% of carbon in NVP@C by TGA	Citric acid, carbon cloth	NVP@C:carbon cloth 20:80	18.4	1M $NaClO_4$ EC:DEC (1:1), 5% FEC	23.3 @ 0.023 14 @ 4.68	[14] Guo et al., Nano Energy 2018

11	NVP@C nanoflake spheres; 9.72% of carbon	Sodium oleate	NVP@C:CB: PVDF 75:15:10	67.7	1M $NaClO_4$ PC:FEC (95:5)	77.6 @ 0.047<br>48 @ 9.41	[15] Cao et al., *Nano Energy* 2019
12	NVP@C 3D, nanoflakes; 10.53% carbon	Span 80, paraffin	NVP@C:CB:PVDF 75:15:10	67.1	1M $NaClO_4$ in PC:FEC (95:5)	85.7 @ 0.044<br>65.8 @ 8.82	[16] Zhao et al., *Chem. Eng. J.* 2018
13	NVP-C, 7% of carbon by TGA	Sugar	NVP@C:CB:PVDF 70:18:12	64.4	1M $NaPF_6$ in diglyme	72.8 @ 0.0036<br>60.9 @ 0.36	[17] Pandit et al., Nano Energy 2022
14	NVPF/NVP@C Carbon content in AM is n.a.	Citric acid	NVPF/NVP @C:CB:PVDF 70:20:10	≤70	1M $NaPF_6$ in diglyme	80.3 @ 0.045<br>61.6 @ 0.9	[18] Park et al., *J Mater. Chem. A* 2020
15	NVNiP-F2@C 6.27% of carbon by EDS	Citric acid	NVNiP-F2@C:CB:PVDF 80:10:10	75	1M $NaPF_6$ in EC:PC (1:1)	93.6 @ 0.01<br>84 @ 0.51	[19] Essehli et al., *Adv. Sci.* 2023
16	NVMNP@C, 2.34% of C by TGA	Citric acid	NVMNP@C: CNTs:PVDF 70:20:10	68.4	1M $NaClO_4$ in PC:EC with 5% FEC	54.6 @ 0.05<br>46.9 @ 3	[20] Xu et al., *Small Struct.* 2024
17	NVFP-650@C 6.5% of C by TGA	Oxalic acid	NVFP:CB:PVDF 70:20:10	67.2	1M $NaClO_4$ solvent n.a.	72 @ 0.007<br>47.5 @ 1.4	[21] Zhu et al., *Small Methods* 2025
18	$Na_3V_2O_2(PO_4)_2F$, C content n.a.	Oxalic acid	AM:CB:PVDF 60:30:10	≤60	1 M $NaClO_4$ in PC, 5% FEC	81.4 @ 0.0078<br>68.4 @ 0.78	[22] Zheng et al., *Appl Mater Today* 2021
19	NVPOF, no carbon shell	Oxalic acid	NVPOF-80:CB:CMC 70:20:10	70	1M $NaClO_4$ in EC:PC (1:1), 5% FEC	87.2 @ 0.091<br>48 @ 9.1	[23] Yu et al., *Sci Bull* 2025
20	2%PANI@NVPF, 2.63% of carbon by TGA	Citric acid, PANI	2%PANI@NVPF:CB:PVDF 70:20:10	68.2	1M $NaClO_4$ in PC:FEC (95:5%)	78.8 @ 0.045<br>59.5 @ 0.45	[24] Missaoui et al., ACS AMI 2024
21	NVAMP-0.3 3.43% of carbon by TGA	Oxalic acid, citric acid	AM:CB:PVDF 7:2:1	67.6	1 M $NaClO_4$ EC:PC (1:1) 5%FEC	65.8 @ 0.07<br>61.4 @ 2.8	[25] Liu et al., *Chem Eng J* 2024
22	NV_2O/NV_3P@C@SWCNTs, ~6% SWCNTs (in reaction mixture)	Citric acid, SWCNTs	NV_2O/NV_3P@C@ SWCNTs:CB:PVDF 8:1:1	~75.2	1M $NaClO_4$ EC:PC (1:1), 5% FEC	84.2 @ 0.047<br>55.2 @ 9.4	[26] Wang et al., *J. Mater. Sci. Technol.* 2021
23	NVPFO/MCLC@C 4.6 % of carbon by TGA	Melamine, cyanuric and citric acid, alkali lignin	NVPFO/MCLC@C CB:PVDF 8:1:1	76.3	1M $NaClO_4$ in EC:PC (1:1), 5%FEC	93.8 @ 0.1<br>42.24 @ 10.0	[27] Ma et al., *Chem Eng J* 2024
24	NVOPF, zero carbon content	Citric acid	NVOPF:CB:PTFE 7:2:1	70.0	1M $NaClO_4$ in PC, 2 %FEC	87.4 @ 0.0091<br>69.9 @ 1.82	[28] Li et al., Adv Mater 2024
25	NVP@C, 20.67% of carbon based on TGA	Citric acid	NVP@C:CB:PVDF 8:1:1	63.7	1M $NaClO_4$ in EC:DEC (1:1)	79.2 @ 0.047<br>54.6 @ 0.936	[29] Kate et al., *J Energy Storage* 2023

*EM-specific parameters and NVP content were derived from the publications' data:

$$\text{NVP in EM (\%)} = \omega_{\text{NVP@C in EM (\%)}} \times (100 - \omega_{\text{C in NVP@C (\%)}})$$

where $\omega_{C\ in\ NVP@C}$ is the reported content of carbon in NVP@C active material or change of mass in TGA in the 100°C to 630°C temperature range;

$Q_{EM}(\text{mAh } g_{EM}^{-1}) = Q_{AM}\ (\text{mAh } g_{AM}^{-1}) \times \omega_{AM}$

where ω_{AM} is mass fraction of NVP@C active material in electrode material;

$I_{EM}(\text{A } g_{EM}^{-1}) = I_{AM}\ (\text{A } g_{AM}^{-1}) \times \omega_{AM}$;

For C-rate to specific current recalculation theoretical capacity was used assuming NVP@carbon shell as the AM.

Table S5. Electrochemical impedance spectroscopy fitting parameters for NVP@GA cathode.

SOC (%)	R_b (Ω cm^{-2})	R_{CT1} (Ω cm^{-2})	Q_1 (S s^n)	n_1	R_{CT2} (Ω cm^{-2})	Q_2 (S s^n)	n_2	W (S s$^{0.5}$)	Std. Error (%)
0	1.49	13.5	3.09×10^{-4}	0.709	-	4.0×10^{-2}	1	0.029	2.8
11	1.58	4.6	3.69×10^{-4}	0.709	0.7	1.1×10^{-2}	1	0.347	1.5
22	1.59	3.8	3.16×10^{-4}	0.727	0.8	1.3×10^{-2}	1	0.495	1.3
33	1.60	3.3	3.02×10^{-4}	0.736	0.8	1.3×10^{-2}	1	0.551	1.3
44	1.61	2.9	3.02×10^{-4}	0.741	0.9	1.3×10^{-2}	1	0.586	1.3
56	1.61	2.6	3.26×10^{-4}	0.737	0.9	1.5×10^{-2}	1	0.625	1.3
67	1.61	2.3	3.02×10^{-4}	0.731	1.0	1.5×10^{-2}	1	0.596	1.2
78	1.61	2.1	3.97×10^{-4}	0.728	1.1	1.6×10^{-2}	1	0.443	1.4
89	1.61	1.9	4.39×10^{-4}	0.725	1.0	1.5×10^{-2}	1	0.192	1.2
100	1.61	1.6	4.55×10^{-4}	0.732	-	7.5×10^{-2}	0.976	0.183	1.9

Supplementary References

(1) Tuinstra, F.; Koenig, J. L. Raman Spectrum of Graphite. *J. Chem. Phys.* **1970**, *53* (3), 1126–1130. https://doi.org/10.1063/1.1674108.

(2) Sadezky, A.; Muckenhuber, H.; Grothe, H.; Niessner, R.; Pöschl, U. Raman Microspectroscopy of Soot and Related Carbonaceous Materials: Spectral Analysis and Structural Information. *Carbon* **2005**, *43* (8), 1731–1742. https://doi.org/10.1016/j.carbon.2005.02.018.

(3) Forghani, M.; Donne, S. W. Method Comparison for Deconvoluting Capacitive and Pseudo-Capacitive Contributions to Electrochemical Capacitor Electrode Behavior. *J. Electrochem. Soc.* **2018**, *165* (3), A664. https://doi.org/10.1149/2.0931803jes.

(4) Wen, C. J.; Boukamp, B. A.; Huggins, R. A.; Weppner, W. Thermodynamic and Mass Transport Properties of " LiAl ." *J. Electrochem. Soc.* **1979**, *126* (12), 2258. https://doi.org/10.1149/1.2128939.

(5) Weppner, W.; Huggins, R. A. Determination of the Kinetic Parameters of Mixed-Conducting Electrodes and Application to the System Li3Sb. *J. Electrochem. Soc.* **1977**, *124* (10), 1569. https://doi.org/10.1149/1.2133112.

(6) Hobold, G. M.; Kim, K.-H.; Gallant, B. M. Beneficial vs. Inhibiting Passivation by the Native Lithium Solid Electrolyte Interphase Revealed by Electrochemical Li+ Exchange. *Energy Environ. Sci.* **2023**, *16* (5), 2247–2261. https://doi.org/10.1039/D2EE04203G.

(7) Zhou, Q.; Wang, L.; Li, W.; Zeng, S.; Zhao, K.; Yang, Y.; Wu, Q.; Liu, M.; Huang, Q.; Zhang, J.; Sun, X. Carbon-Decorated Na3V2(PO4)3 as Ultralong Lifespan Cathodes for High-Energy-Density Symmetric Sodium-Ion Batteries. *ACS Appl. Mater. Interfaces* **2021**, *13* (21), 25036–25043. https://doi.org/10.1021/acsami.1c06160.

(8) Chen, H.; Zhang, B.; Wang, X.; Dong, P.; Tong, H.; Zheng, J.; Yu, W.; Zhang, J. CNT-Decorated Na3V2(PO4)3 Microspheres as a High-Rate and Cycle-Stable Cathode Material for Sodium Ion Batteries. *ACS Appl. Mater. Interfaces* **2018**, *10* (4), 3590–3595. https://doi.org/10.1021/acsami.7b16402.

(9) Zhu, P.; Li, J.; Wang, Y.; Jin, Y. Multi-Enhanced High-Entropy NASICON Cathodes for High Voltage and Stability in Sodium-Ion Batteries. *ACS Appl. Mater. Interfaces* **2025**, *17* (33), 46967–46976. https://doi.org/10.1021/acsami.5c08297.

(10) Chen, R.; Li, D.; Zhang, X.; Zhang, S.; Wang, X.; Li, S.; Gural'skiy, I. A.; Zatovsky, I. V.; Han, W.; Butenko, D. Synergistic Effect of Ternary Substitution in Na3V2(PO4)3 for High-Rate and Long-Life Anode-Free SIBs. *ACS Appl. Mater. Interfaces* **2025**, *17* (32), 45859–45873. https://doi.org/10.1021/acsami.5c08411.

(11) Xia, F.; Ahangari, M.; Wu, J.; Tran, D. H. H.; Zhou, X.; Chen, Z.; Luo, H.; Zhou, M. Reductive Carbon as an Additive Enables the High Capacity and Durability of NASICON Structured

Sodium-Ion Batteries. *ACS Appl. Energy Mater.* **2025**, *8* (7), 4355–4361. https://doi.org/10.1021/acsaem.4c03244.
(12) Li, Y.; Lai, X.-Q.; Yang, S.-J.; Wang, P.-F.; Liu, Z.-L.; Shu, J.; Yi, T.-F. Unraveling the Function Mechanism of N-Doped Carbon-Encapsulated Na3V2(PO4)3 Cathode toward High-Performance Sodium-Ion Battery with Ultrahigh Cycling Stability. *ACS Appl. Mater. Interfaces* **2025**, *17* (2), 3840–3851. https://doi.org/10.1021/acsami.4c14656.
(13) Wang, C.; Du, D.; Song, M.; Wang, Y.; Li, F. A High-Power Na3V2(PO4)3-Bi Sodium-Ion Full Battery in a Wide Temperature Range. *Adv. Energy Mater.* **2019**, *9* (16), 1900022. https://doi.org/10.1002/aenm.201900022.
(14) Guo, D.; Qin, J.; Yin, Z.; Bai, J.; Sun, Y.-K.; Cao, M. Achieving High Mass Loading of Na3V2(PO4)3@carbon on Carbon Cloth by Constructing Three-Dimensional Network between Carbon Fibers for Ultralong Cycle-Life and Ultrahigh Rate Sodium-Ion Batteries. *Nano Energy* **2018**, *45*, 136–147. https://doi.org/10.1016/j.nanoen.2017.12.038.
(15) Cao, X.; Pan, A.; Yin, B.; Fang, G.; Wang, Y.; Kong, X.; Zhu, T.; Zhou, J.; Cao, G.; Liang, S. Nanoflake-Constructed Porous Na3V2(PO4)3/C Hierarchical Microspheres as a Bicontinuous Cathode for Sodium-Ion Batteries Applications. *Nano Energy* **2019**, *60*, 312–323. https://doi.org/10.1016/j.nanoen.2019.03.066.
(16) Zhao, Y.; Cao, X.; Fang, G.; Wang, Y.; Yang, H.; Liang, S.; Pan, A.; Cao, G. Hierarchically Carbon-Coated Na3V2(PO4)3 Nanoflakes for High-Rate Capability and Ultralong Cycle-Life Sodium Ion Batteries. *Chem. Eng. J.* **2018**, *339*, 162–169. https://doi.org/10.1016/j.cej.2018.01.088.
(17) Pandit, B.; Sougrati, M. T.; Fraisse, B.; Monconduit, L. Exploration of a Na3V2(PO4)3/C –Pb Full Cell Na-Ion Prototype. *Nano Energy* **2022**, *95*, 107010. https://doi.org/10.1016/j.nanoen.2022.107010.
(18) Park, J. Y.; Shim, Y.; Kim, Y.; Choi, Y.; Lee, H. J.; Park, J.; Wang, J. E.; Lee, Y.; Chang, J. H.; Yim, K.; Ahn, C. W.; Lee, C.-W.; Kim, D. K.; Yuk, J. M. An Iron-Doped NASICON Type Sodium Ion Battery Cathode for Enhanced Sodium Storage Performance and Its Full Cell Applications. *J. Mater. Chem. A* **2020**, *8* (39), 20436–20445. https://doi.org/10.1039/D0TA07766F.
(19) Essehli, R.; Yahia, H. B.; Amin, R.; Li, M.; Morales, D.; Greenbaum, S. G.; Abouimrane, A.; Parejiya, A.; Mahmoud, A.; Boulahya, K.; Dixit, M.; Belharouak, I. Sodium Rich Vanadium Oxy-Fluorophosphate – Na3.2Ni0.2V1.8(PO4)2F2O – as Advanced Cathode for Sodium Ion Batteries. *Adv. Sci.* **2023**, *10* (22), 2301091. https://doi.org/10.1002/advs.202301091.
(20) Xu, S.; Zhu, W.; Yang, Y.; Yao, Y.; Ali, G.; Zhang, X.; Rui, X.; Yu, Y. Bimetal-Substituted Polyanion Cathode for Sodium-Ion Batteries: Less Vanadium and Boosted Low-Temperature Kinetics. *Small Struct.* **2024**, *5* (5), 2300369. https://doi.org/10.1002/sstr.202300369.
(21) Zhu, C.; Liu, X.; Li, C.; Chen, Y.; Guo, X.; Luo, D.; Ji, W.; Deng, W.; Li, R. Sodium-Deficient NASICON Na3+xVFe(PO4)3 Cathode for High-Performance Sodium-Ion Batteries. *Small Methods* **2025**, *9* (5), 2401697. https://doi.org/10.1002/smtd.202401697.
(22) Zheng, L.; Zhang, D.; Wang, X.; Guo, G. Continuous-Flow Rapid and Controllable Microfluidic Synthesis of Sodium Vanadium Fluorophosphate as a Cathode Material. *Appl. Mater. Today* **2021**, *23*, 101032. https://doi.org/10.1016/j.apmt.2021.101032.
(23) Yu, H.; Wang, J.; Jing, H.; Wu, C.; Hu, E.; Xi, S.; Wang, X.; Fang, Z.; Wu, X.-L.; Liang, Q.; Qi, W.; Yan, Q.; Wang, H.; Du, C.-F. Broadening the Na+ Diffusion Degree of Freedom to Unlock a Rapid Sodium Storage Potential in Fluorophosphate Cathode. *Sci. Bull.* **2025**. https://doi.org/10.1016/j.scib.2025.06.005.
(24) Missaoui, K.; Ferchichi, K.; Amdouni, N.; Gómez-Cámer, J. L.; Pérez-Vicente, C.; Bonilla, A.; Cosano, D.; Caballero, Á.; Ortiz, G. F. Polyaniline-Coated Na3V2(PO4)2F3 Cathode Enables Fast Sodium Ion Diffusion and Structural Stability in Rechargeable Batteries. *ACS Appl. Mater. Interfaces* **2024**, *16* (38), 50550–50560. https://doi.org/10.1021/acsami.4c05832.
(25) Liu, X.; Zhu, C.; Xu, T.; Li, C.; Guo, X.; Jiang, N.; Chen, Y.; Xu, Y.; Zhu, R.; Zou, W.; Deng, W.; Li, R. Multifunctional-Element Doping of NASICON-Structured Cathode Enables High-Rate and Stable Sodium Storage. *Chem. Eng. J.* **2024**, *497*, 154304. https://doi.org/10.1016/j.cej.2024.154304.

(26) Wang, C.; Long, H.; Zhou, L.; Shen, C.; Tang, W.; Wang, X.; Tian, B.; Shao, L.; Tian, Z.; Su, H.; Xie, K. A Multiphase Sodium Vanadium Phosphate Cathode Material for High-Rate Sodium-Ion Batteries. *J. Mater. Sci. Technol.* **2021**, *66*, 121–127. https://doi.org/10.1016/j.jmst.2020.05.076.

(27) Ma, J.; Zu, X.; Qiu, X.; Zhang, W. Dual Carbon Skeleton Supported NVPFO Sodium-Ion Battery Cathode with High Rate Capability, Superior Cycling Performance, and Wide Temperature Adaptability. *Chem. Eng. J.* **2024**, *493*, 152792. https://doi.org/10.1016/j.cej.2024.152792.

(28) Li, S.; Lu, X.; Li, Y.; Wang, H.; Sun, Y.; Zhou, Q.; Yue, J.; Guo, R.; Wu, F.; Wu, C.; Bai, Y. Dynamic Lock-And-Release Mechanism Enables Reduced ΔG at Low Temperatures for High-Performance Polyanionic Cathode in Sodium-Ion Batteries. *Adv. Mater.* **2024**, *36* (49), 2413013. https://doi.org/10.1002/adma.202413013.

(29) Kate, R. S.; Kadam, S. V.; Kulkarni, M. V.; Deokate, R. J.; Kale, B. B.; Kalubarme, R. S. Highly Stable and Nanoporous Na3V2(PO4)3@C Cathode Material for Sodium-Ion Batteries Using Thermal Management. *J. Energy Storage* **2023**, *74*, 109245. https://doi.org/10.1016/j.est.2023.109245.
